# A Mechanistic Study on the Formation of Dronic Acids

**DOI:** 10.3390/molecules26247587

**Published:** 2021-12-14

**Authors:** Péter Ábrányi-Balogh, István Greiner, György Keglevich

**Affiliations:** 1 Research Centre for Natural Sciences, Medicinal Chemistry Research Group, 1117 Budapest, Hungary; 2 Gedeon Richter Plc., 1475 Budapest, Hungary; i.greiner@richter.hu; 3 Department of Organic Chemistry and Technology, Budapest University of Technology and Economics, 1521 Budapest, Hungary

**Keywords:** dronic acid, carboxylic acid, phosphorus trichloride, phosphorous acid, solvents, mechanism, calculations

## Abstract

Dronic acid derivatives, important drugs against bone diseases, may be synthesized from the corresponding substituted acetic acid either by reaction with phosphorus trichloride in methanesulfonic acid as the solvent or by using also phosphorous acid as the P-reactant if sulfolane is applied as the medium. The energetics of the two protocols were evaluated by high-level quantum chemical calculations on the formation of fenidronic acid and benzidronic acid. The second option, involving (HO)_2_P-O-PCl_2_ as the nucleophile, was found to be more favorable over the first variation, comprising Cl_2_P-O-SO_2_Me as the real reagent, especially for the case of benzidronate.

## 1. Introduction

**Figure 1:**
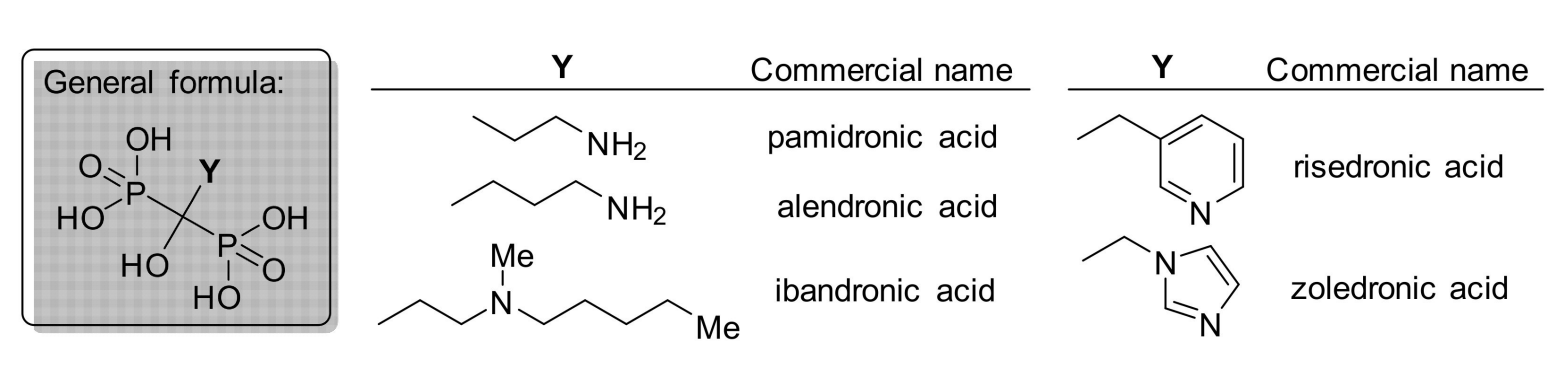
*N*-containing dronic acid derivatives.

Hydroxymethylenebisphosphonic (dronic) acid derivatives include important representatives that are used in the treatment of bone illnesses, such as osteoporosis, the Paget disease, and tumor-induced hypercalcemia [1,2,3,4]. The first-generation agents (etidronate, clodronate, and tiludronate) were only moderately potent, allowing a narrow therapeutic window regarding the desired inhibition and impairment of bone mineralization. Hence, they were replaced by second-generation (*N*-aliphatic) drugs, such as pamidronate, alendronate, and ibandronate [5,6,7], and third-generation (*N*-heterocyclic) species, such as risedronate and zoledronate, in clinical use [8,9]. The most important derivatives are shown in Figure 1.

The general method for the synthesis of dronic acid derivatives involves the reaction of the corresponding substituted acetic acid with phosphorus trichloride (PCl_3_) or with PCl_3_ and phosphorous acid (P(OH)_3_) in different solvents. The optimum conditions (molar ratios, solvent, temperature, and reaction time) as well as the role of the reagents and the mechanism were not described. Moreover, the purity of the products was not clarified and hence misleading yields were published. Keglevich, Grün, and Greiner, together with co-workers, investigated the chemistry of dronic derivatives in detail [4]. The optimum conditions were explored on the basis of the putative mechanisms substantiated by us [10]. According to this, for carrying out the reactions in methanesulfonic acid (MSA), there was the need for 3–3.2 equivalents of the PCl_3_. It was assumed that 1 equivalent of the PCl_3_ converts the substituted acetic acid to the corresponding chloride, while 2 equivalents are needed to provide the two P-functions. Both PCl_3_ and Cl_2_P-O-SO_2_Me (formed from PCl_3_ and MSA) were assumed to be the nucleophiles [10]. At the same time, applying sulfolane as the solvent, there was a need for at least 2 equivalents of both PCl_3_ and P(OH)_3_. In this case, Cl_2_P-O-P(OH)_2_ was found to be the nucleophile [10].

Results of the synthesis of the most important dronic acid derivatives applying the above-mentioned methods (PCl_3_/MSA or PCl_3_/H_3_PO_3_/sulfolane) are summarized in Table 1. On the basis of the diverse yields of the two methods, it is not possible to judge which one is to be preferred. It was, however, unambiguous that using sulfolane, a 2:3 or 2:4 ratio of PCl_3_ and P(OH)_3_, was in most cases advantageous.

As, with a few exceptions, the preparative results of the two methods for the different dronic acid derivatives were not comparable due to unreliable experiments, we wished to evaluate the energetics of the dronate formation on simple models using quantum chemical calculations. The two models selected were the formation of fenidronate and benzidronate from benzoic acid and phenylacetic acid, respectively, or from their derivatives.

## 2. Results and Discussion

### 2.1. A Study on the Formation of Fenidronic Acid

**Scheme 1:**
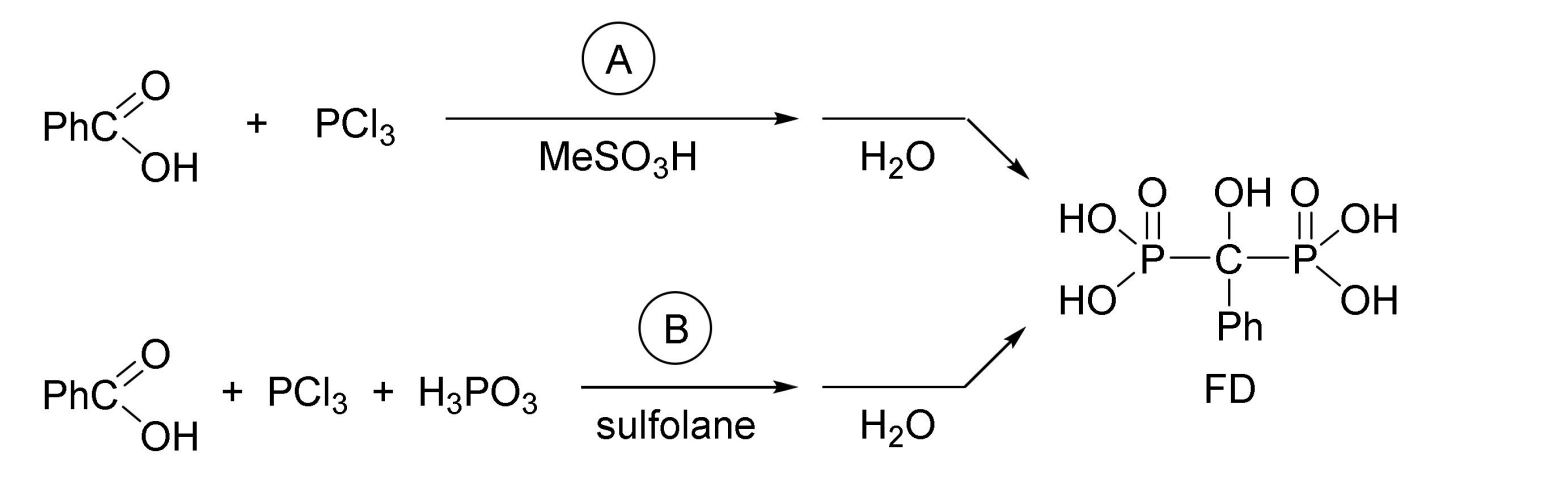
Two models for the theoretical computations on fenidronic acid (FD).

To have a simple model for the calculations, the reaction of benzoic acid with P-reagents was chosen. Version A involved the reaction with PCl_3_ in MSA. Preparative experiments showed that the reaction of benzoic acid with 3.2 equivalents of PCl_3_ in MSA at 75 °C/day, followed by hydrolysis at 105 °C/4 h, pH adjustment to 1.8 by NaOH/H_2_O, and precipitation with methanol afforded the disodium salt of fenidronic acid (fenidronate) in a 46% yield, in a pure form [19]. Version B for the theoretical study comprised the reaction of benzoic acid with PCl_3_ and P(OH)_3_ in sulfolane (Scheme 1).

**Scheme 2:**
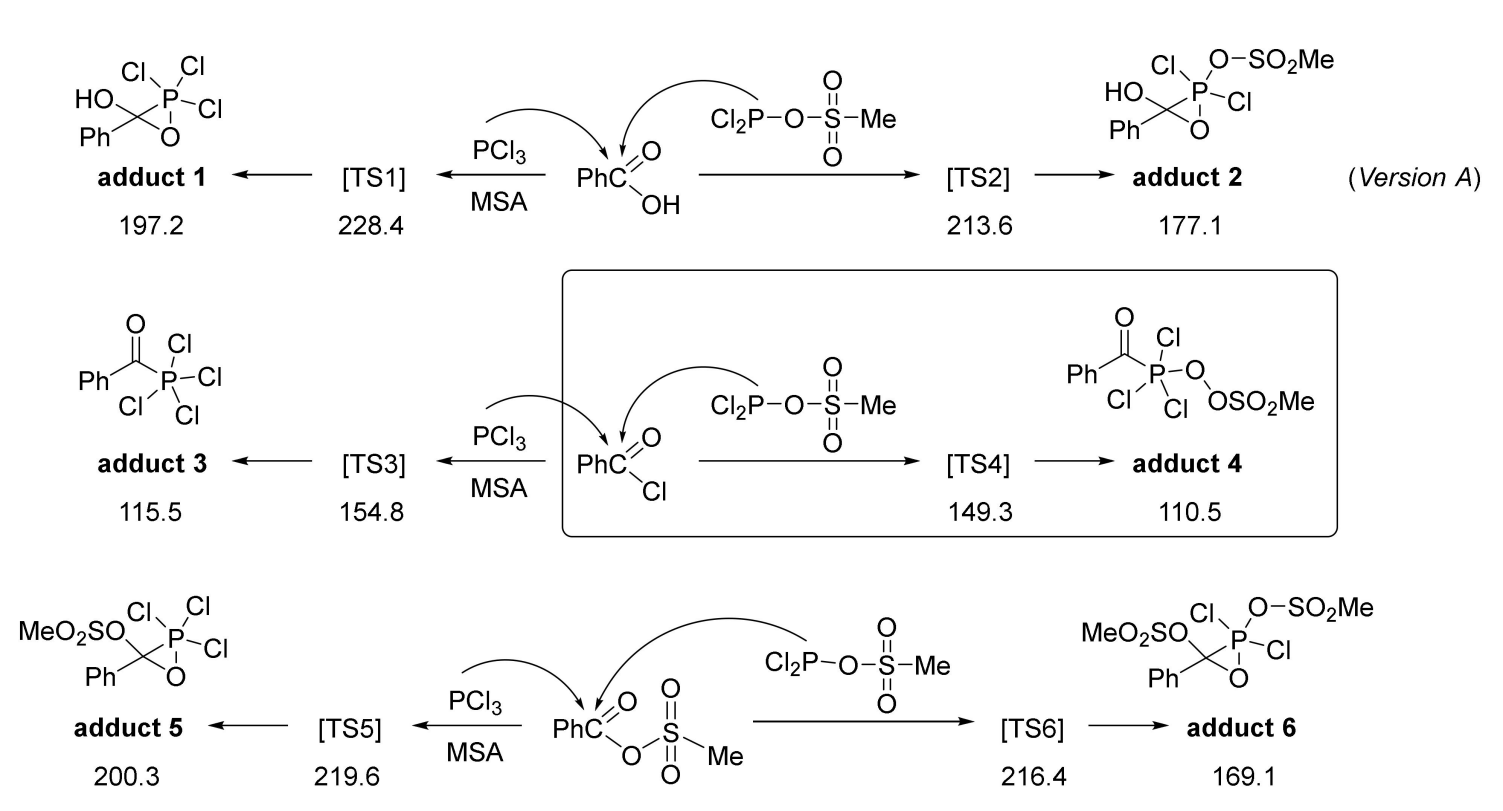
Possible starting steps in the reaction of benzoic acid or its derivatives with PCl_3_ in MSA (the numbers below the TSs mean the corresponding Gibbs free energy of activation).

Applying 3 equivalents of PCl_3_ in MSA, benzoic acid itself, benzoyl chloride formed from the benzoic acid, and the mixed anhydride formed from benzoic acid and methanesulfonyl chloride or from benzoyl chloride and MSA, under the conditions of the reaction may be regarded as the starting substrates undergoing addition of the P-nucleophile. The attacking P-reagents may be PCl_3_ itself and the Cl_2_P-O-SO_2_Me species formed from PCl_3_ and MSA. The variations outlined are summarized in Scheme 2. The anhydrides were assumed already in the earlier stage of our work [20]. We performed DFT-level computations at the M062X/6-311+G (d,p) level considering the solvent effect of water or DMSO simulating MSA and sulfolane, respectively, using the SMD solvent model with the Gaussian 09 program package. One can see that the most advantageous reaction variations were the ones starting from benzoyl chloride and Cl_2_P-O-SO_2_Me or PCl_3_ as the P-nucleophiles (for the best case, see the framed part of Scheme 2). In any other case, the transition states (**TS1,2,5,6**) required an energy investment of higher than 200 kJ mol^−1^ and these reactions were more endothermic. It is also noted that starting from all the three benzoic acid derivatives, irrespective of whether the nucleophile is PCl_3_ or Cl_2_P-O-SO_2_Me, the intermediates contain a pentavalent pentacoordinated phosphorus atom. At the same time, the intermediate formed from benzoic acid or from the mixed anhydride is presumed to incorporate a three-membered oxaphosphirane ring.

**Scheme 3:**
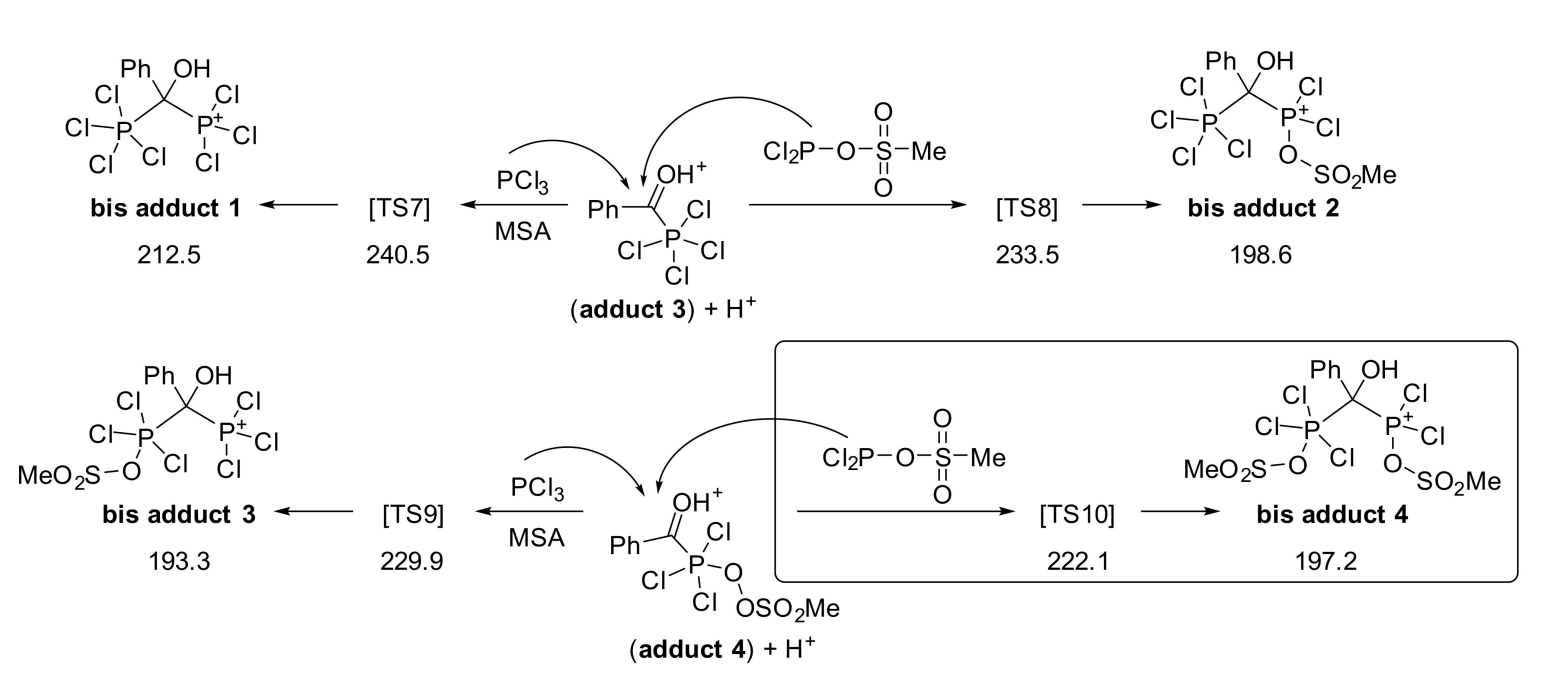
Possible steps for the formation of **bis adducts 1–4** (the numbers below the TSs mean the corresponding Gibbs free energy of activation).

**Figure 2:**
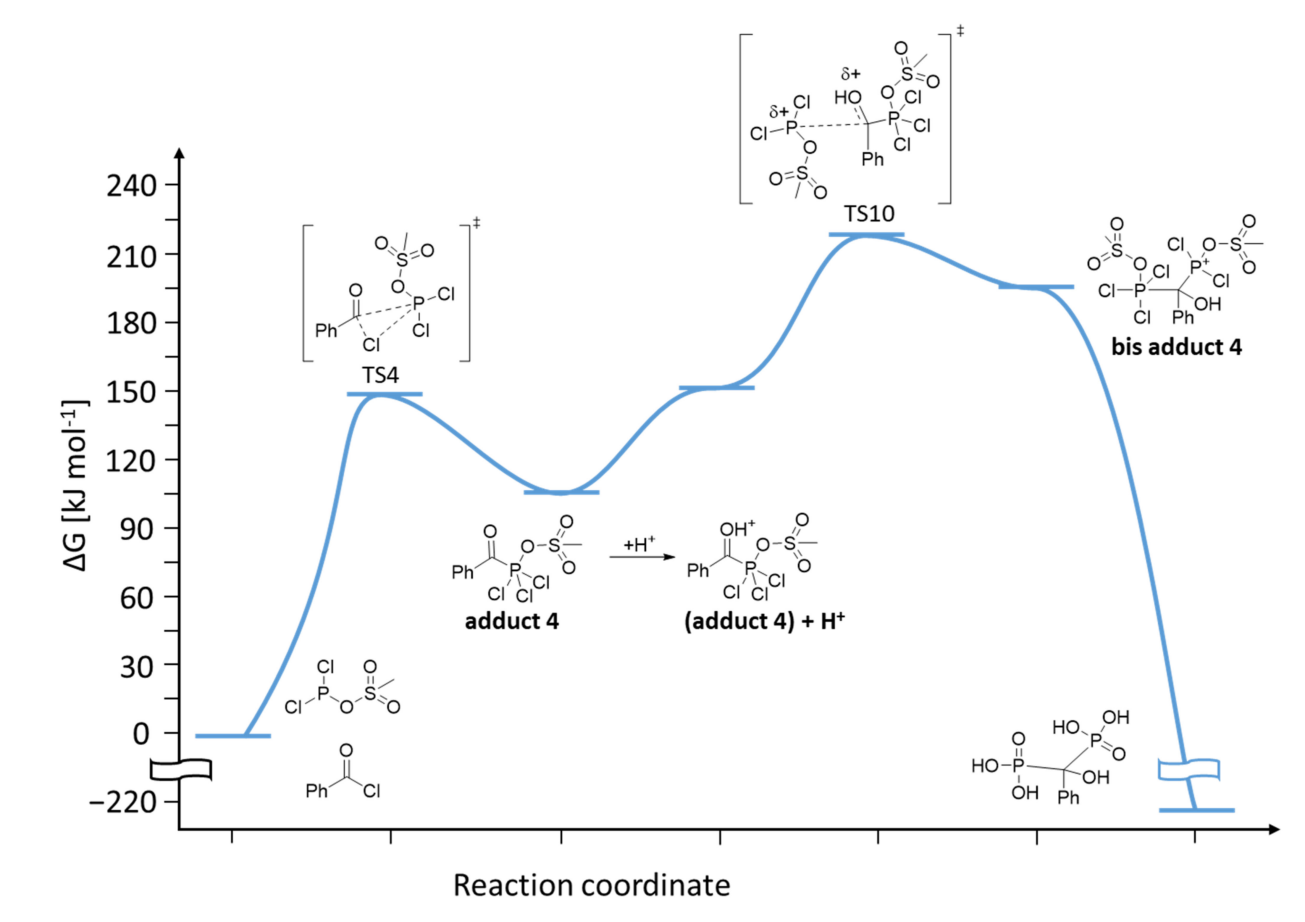
Gibbs free energy diagram for the formation of fenidronic acid from benzoyl chloride and Cl_2_P-O-SO_2_Me.

For the formation of the bis adducts, we chose the energetically most favorable **adduct 3** and **adduct 4** as the starting intermediates. The protonation of these **adducts 3** and **4** on the carbonyl oxygen seemed crucial, as without protonation, no reaction could be observed computationally. It was assumed that the MSA applied in large excess is responsible for the protonation. For the estimation of the protonation energy, we took the gas-phase Gibbs free energy of the proton [21] and took into account the computed Gibbs free energies of MSA and its conjugate base, methanesulfonate. The protonated intermediates ((**adduct 3**) + H^+^ and (**adduct 4**) + H^+^) were then reacted with PCl_3_ or with Cl_2_P-O-SO_2_Me (Scheme 3). The transition states (**TS7**–**TS10**) needed a further 85–98 kJ mol^−1^ energy investment, as compared to the energies of the protonated adducts, and the reactions were endothermic, leading to **bis adducts 1–4**. The reaction with the lowest activation energy (**TS10**) was the one between **adduct 4** and Cl_2_P-O-SO_2_Me, leading to **bis adduct 4** (see the framed part of Scheme 3). Thereafter, the formation of fenidronic acid from **bis adducts 1–4** was calculated. The elimination of HCl and/or MSA on the addition of water took place with an energy release of -390–420 kJ mol^−1^. This means that the final hydrolysis is highly exothermic and might be the driving force for the whole reaction sequence. We have depicted the energetically most advantageous reaction pathway in Figure 2.

**Scheme 4:**
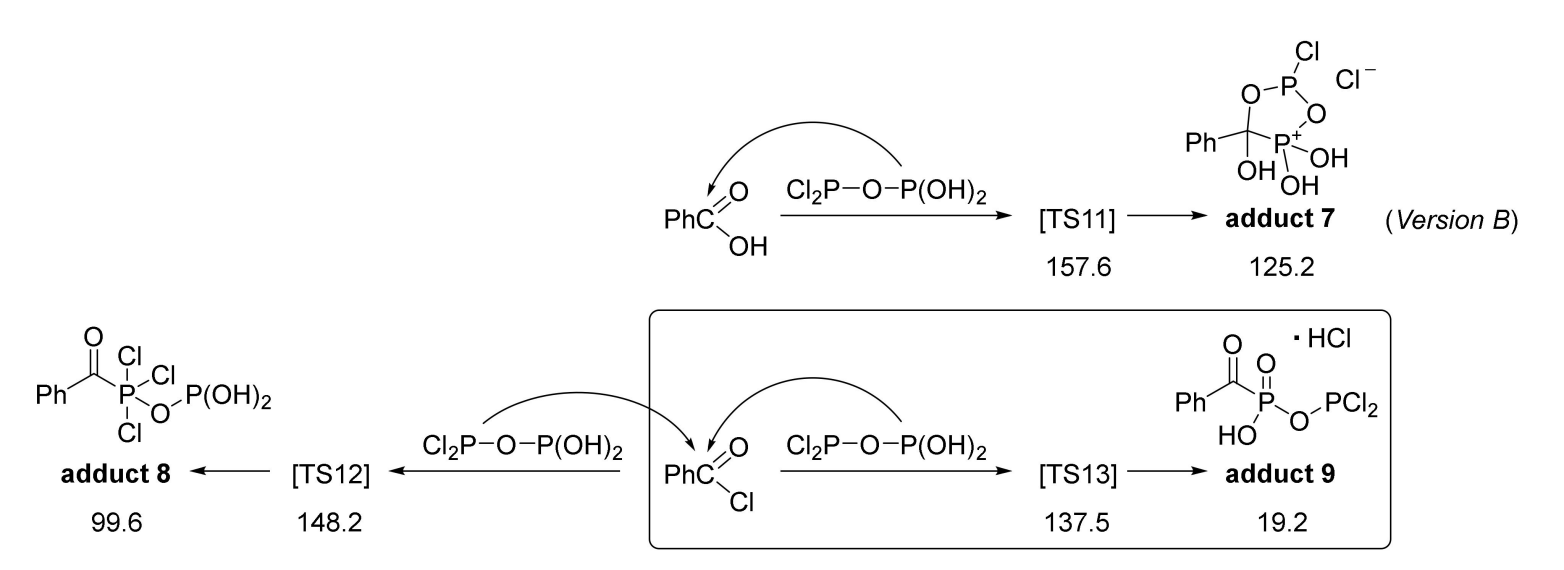
Possible starting steps in the reaction of benzoic acid (or benzoyl chloride), PCl_3_, and P(OH)_3_ in sulfolane (the numbers below the TSs mean the corresponding Gibbs free energy of activation.).

The possible starting materials were assumed to be 2–2 equivalents of PCl_3_ and P(OH)_3_ in sulfolane, benzoic acid, and benzoyl chloride. As regards the P-nucleophile, Cl_2_P-O-P(OH)_2_ formed from PCl_3_ and P(OH)_3_ was regarded as the active agent. At the same time, (HO)_2_P-PCl-P(OH)_2_ could also be the nucleophile. Moreover, as 1 equivalent of PCl_3_ covers the formation of PhC(O)Cl from PhC(O)OH, the involvement of the latter P-nucleophile is more probable. However, to simplify the situation, Cl_2_P-O-P(OH)_2_ was assumed in the calculations. A part of the possibilities mentioned can be seen in Scheme 4. The lowest Gibbs free energy belonged to the transition state of the reaction of benzoyl chloride and Cl_2_P-O-P(OH)_2_ (**TS13**), which resulted in the less endothermic **adduct 9** among the adducts in the series (see the framed part of Scheme 4). **Adduct 9** is formed by P-C addition and HCl elimination in one single step. On the departure of the HCl molecule from the reaction complex, the adduct is stabilized by further −20.2 kJ mol^−1^. It is also seen that the -P(OH)_2_ moiety of the Cl_2_P-O-P(OH)_2_ species is more nucleophilic than the -PCl_2_ unit.

**Scheme 5:**
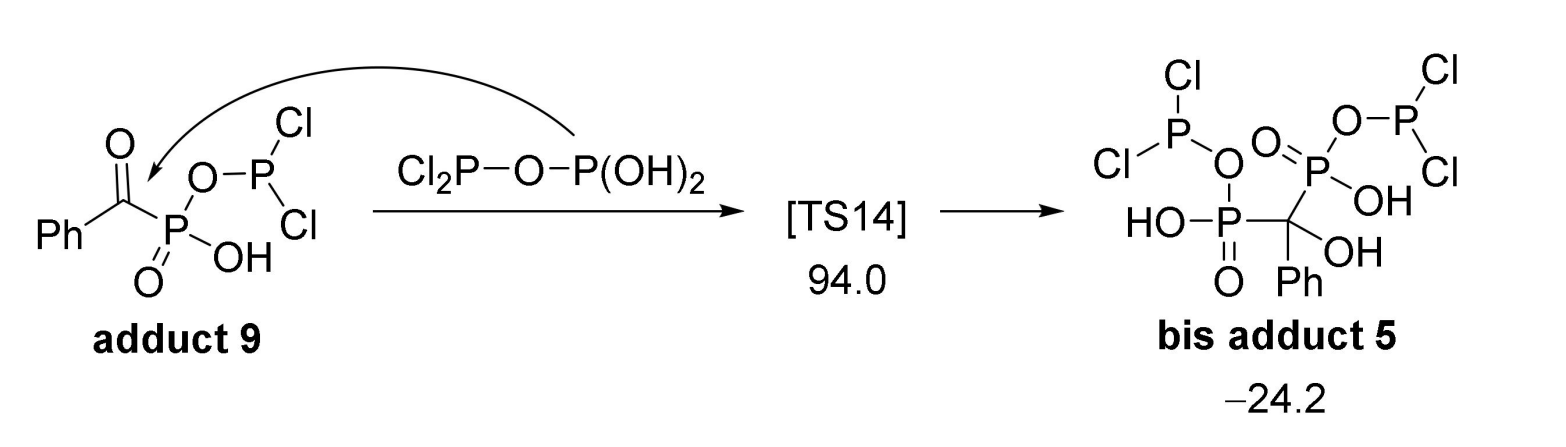
Possible bis adduct forming reaction (the numbers below the TSs mean the corresponding Gibbs free energy of activation).

**Figure 3:**
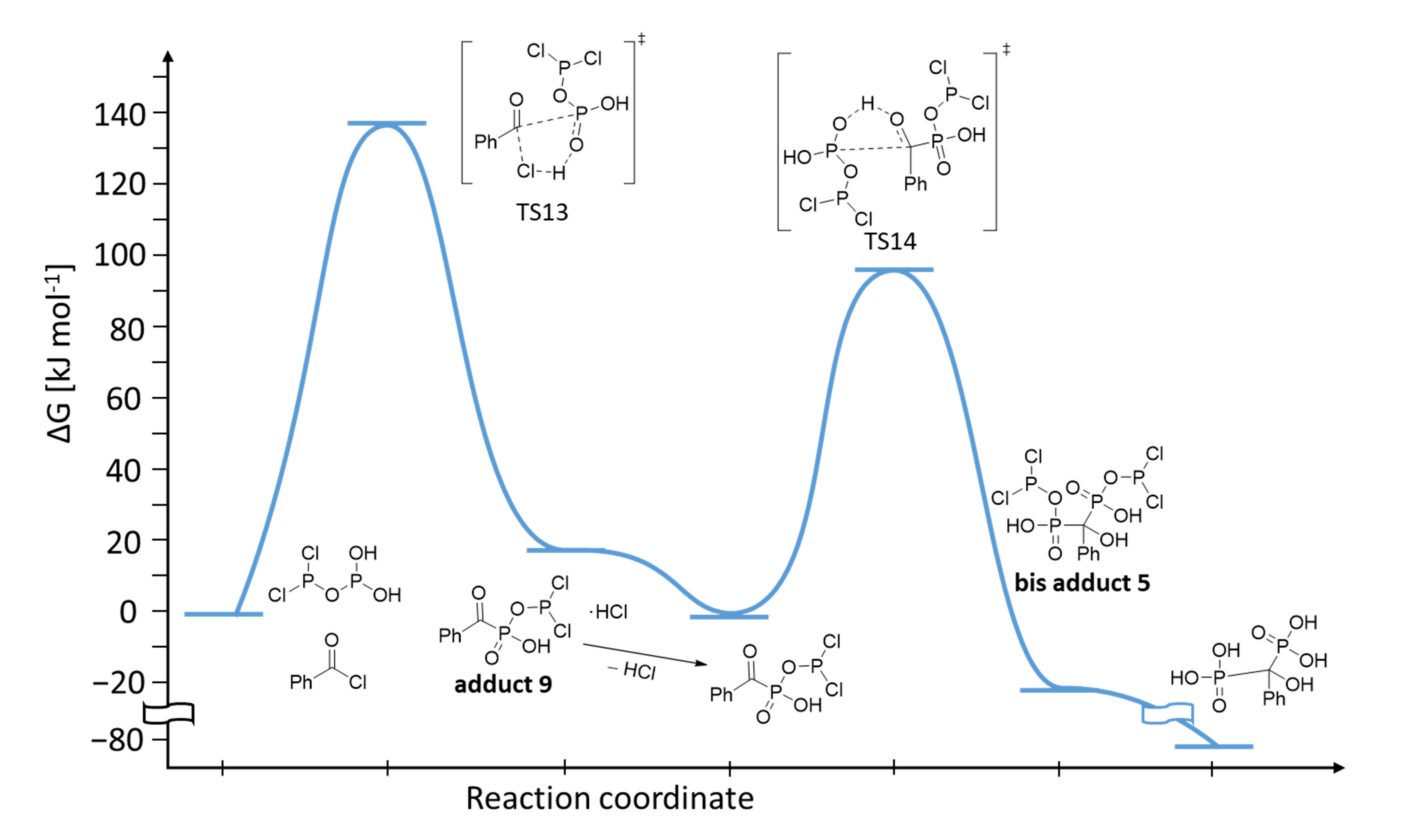
Gibbs free energy diagram for the formation of fenidronic acid from benzoyl chloride and Cl_2_P-O-P(OH)_2_.

The next adduct computed was formed from **adduct 9** and a second molecule of Cl_2_P-O-P(OH)_2_. An important P-OH---O=P hydrogen bridge was present in **TS14**, with an activation energy of 94.0 kJ mol^−1^, leading to **bis adduct 5** in an exothermic reaction (Scheme 5). Again, the final step in this series was the exothermic hydrolysis. The energetically most advantageous reaction pathway is depicted in Figure 3.

### 2.2. A Study on the Formation of Benzidronic Acid

**Scheme 6:**
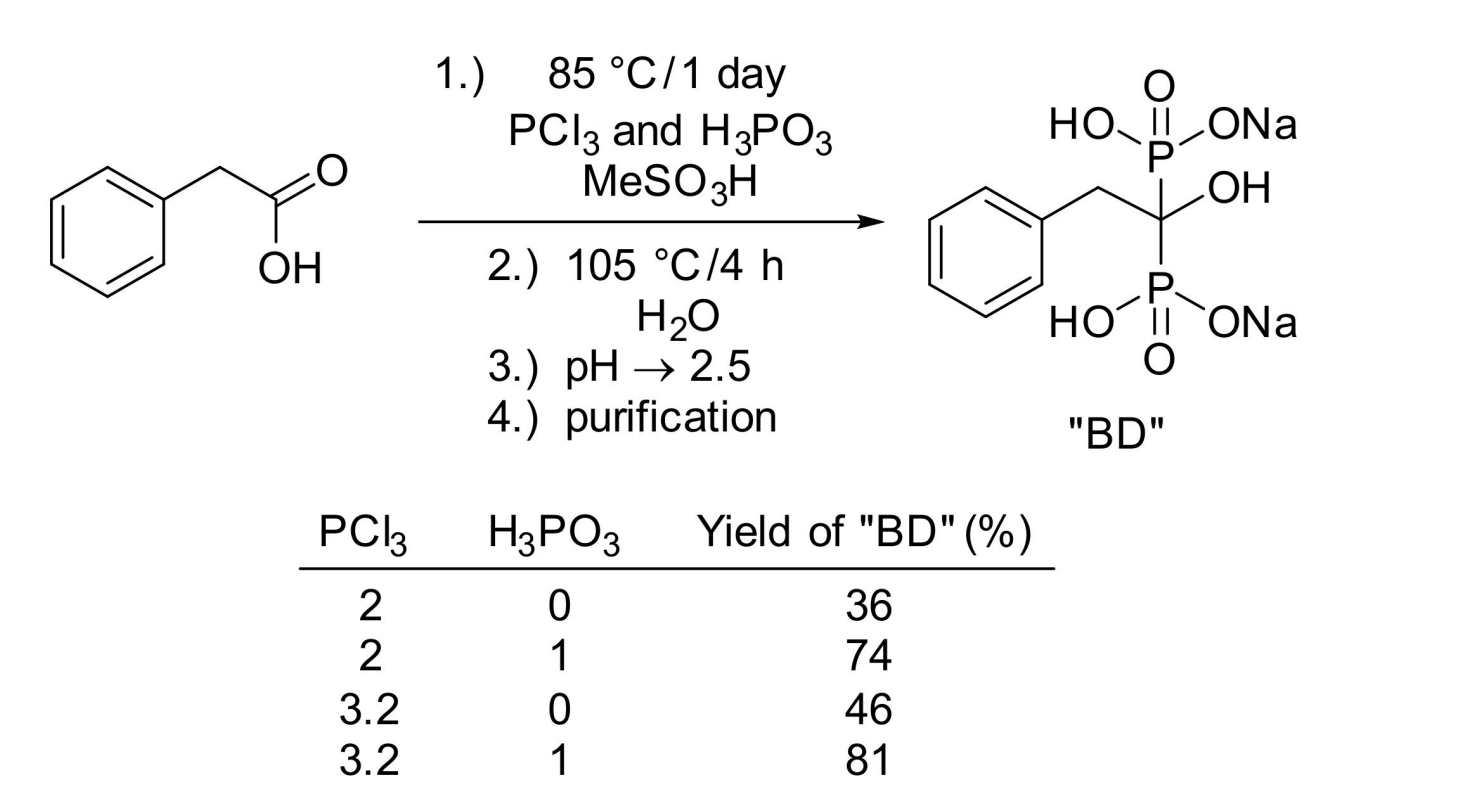
Preparative results for the synthesis of benzidronate (BD) [17].

After investigating the formation of fenidronic acid, we turned our attention to benzidronic acid. The use of 2 equivalents of PCl_3_ gave benzidronate in a yield of 36% (Scheme 6). Further adding 1 equivalent of H_3_PO_3_, the yield increased to 74%. Applying 3.2 equivalents PCl_3_ led to a yield of 46%. When there was also 1 equivalent of H_3_PO_3_, the yield was 81% [22]. The positive effect of H_3_PO_3_ was surprising. The formation of the (HO)_2_P-O-PCl_2_ intermediate must be assumed. In MSA as the solvent, there is only a slight chance that PCl_3_ will react with H_3_PO_3_ to provide (HO)_2_P-O-PCl_2_, as due to the excess of MSA, the probability of the formation of MeSO_2_-O-PCl_2_ is greater. However, the phenylacetyl derivatives may be more capable of reacting with (HO)_2_P-O-PCl_2_ present only in a low concentration.

**Scheme 7:**
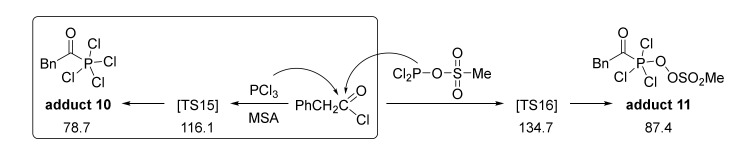
Possible starting steps in the reaction of phenylacetyl chloride and PCl_3_ in MSA (the numbers below the TSs mean the corresponding Gibbs free energy of activation).

During the calculations, we took into consideration phenylacetyl chloride as the starting component reacting with PCl_3_ or with Cl_2_P-O-O_2_SMe in MSA (Scheme 7). The transition states (**TS15** and **TS16**) were of 116.1 and 134.7 kJ mol^−1^, respectively. The values were similarly low as in the reaction with benzoyl chloride, although in this case, the route involving PCl_3_ was the more favorable option (see the framed part of Scheme 7).

**Scheme 8:**
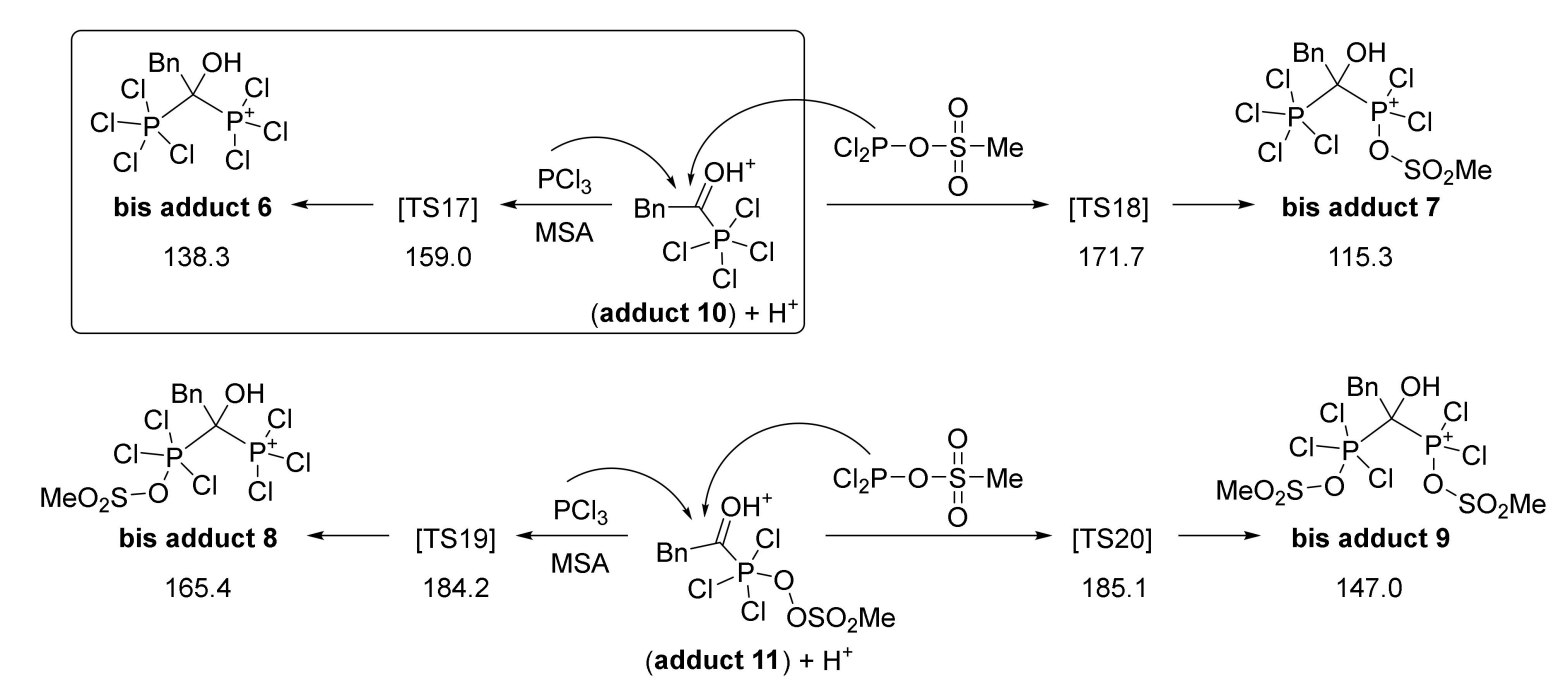
Possible steps for the formation of **bis adducts 6–9** (the numbers below the TSs mean the corresponding Gibbs free energy of activation).

**Figure 4:**
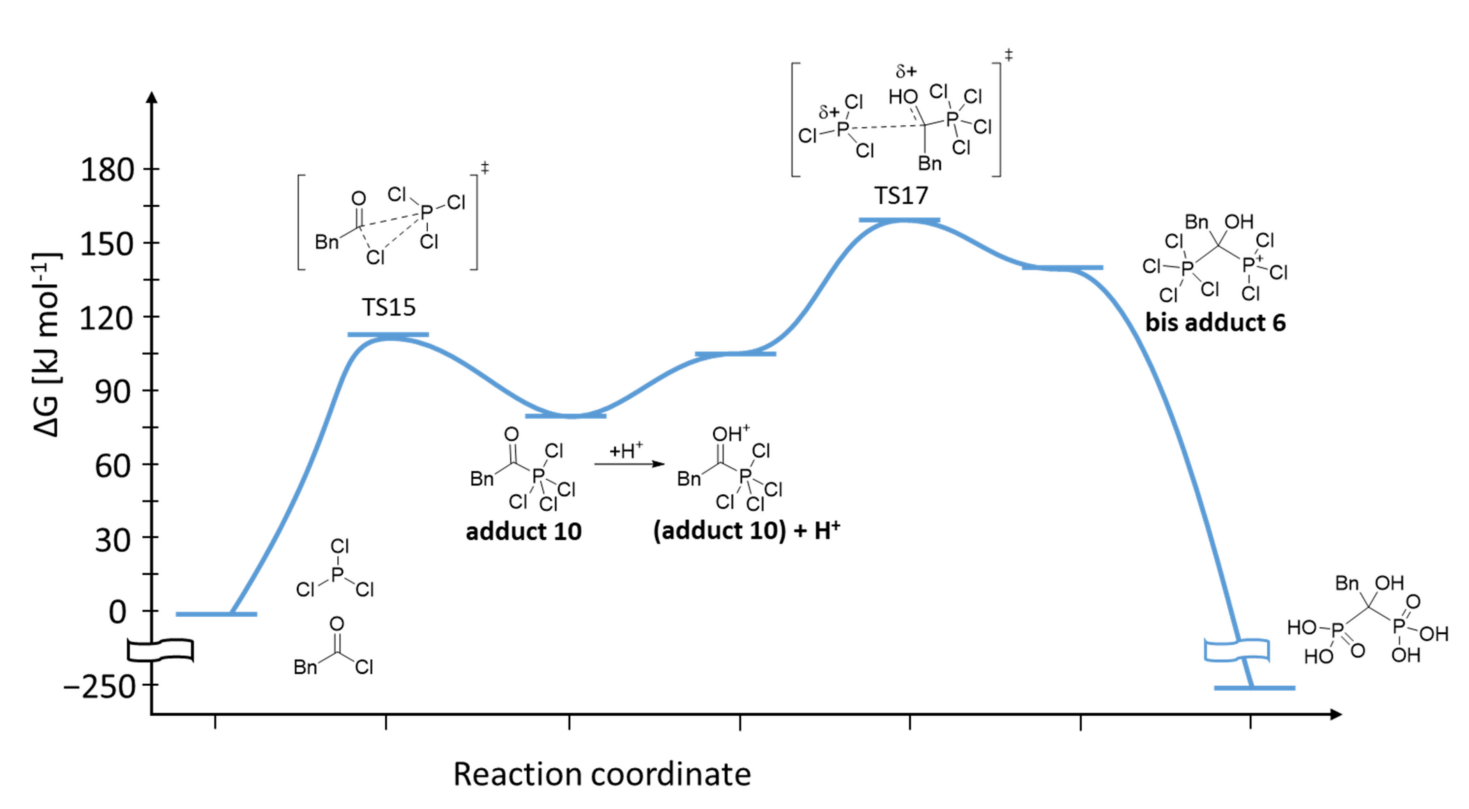
Gibbs free energy diagram for the formation of benzidronic acid from phenylacetyl chloride and PCl_3_.

Both energetically similar pathways were calculated further. The protonation and formation of the corresponding bis adducts from **adducts 10** and **11** was computed assuming both PCl_3_ and Cl_2_P-O-O_2_SMe as the nucleophiles (Scheme 8). Transition states **TS17**–**TS20** required less (54–71 kJ mol^−1^) further energy investment than those in the analogous steps of fenidronic acid formation, and **bis adducts 6–9** had much lower energy levels (between 115 and 165 kJ mol^−1^) as compared to the range of 193–212 kJ mol^−1^ obtained for **bis adducts 1–4** (Scheme 3). In this case, again PCl_3_ was the most favorable reagent (see the framed part of Scheme 8). Notably, taking a look over the energy value of the final dronic acids, one may conclude that the last hydrolysis is again a highly exothermic step (−390–420 kJ mol^−1^). We have depicted the most advantageous reaction pathway in Figure 4.

**Scheme 9:**
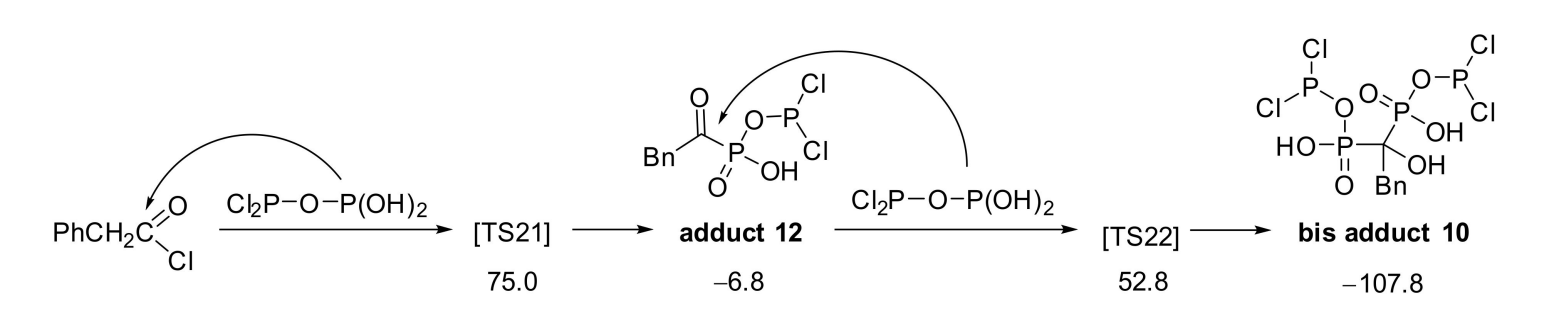
Possible steps for the formation of **bis adduct 10** (the numbers below the TSs mean the corresponding Gibbs free energy of activation).

**Figure 5:**
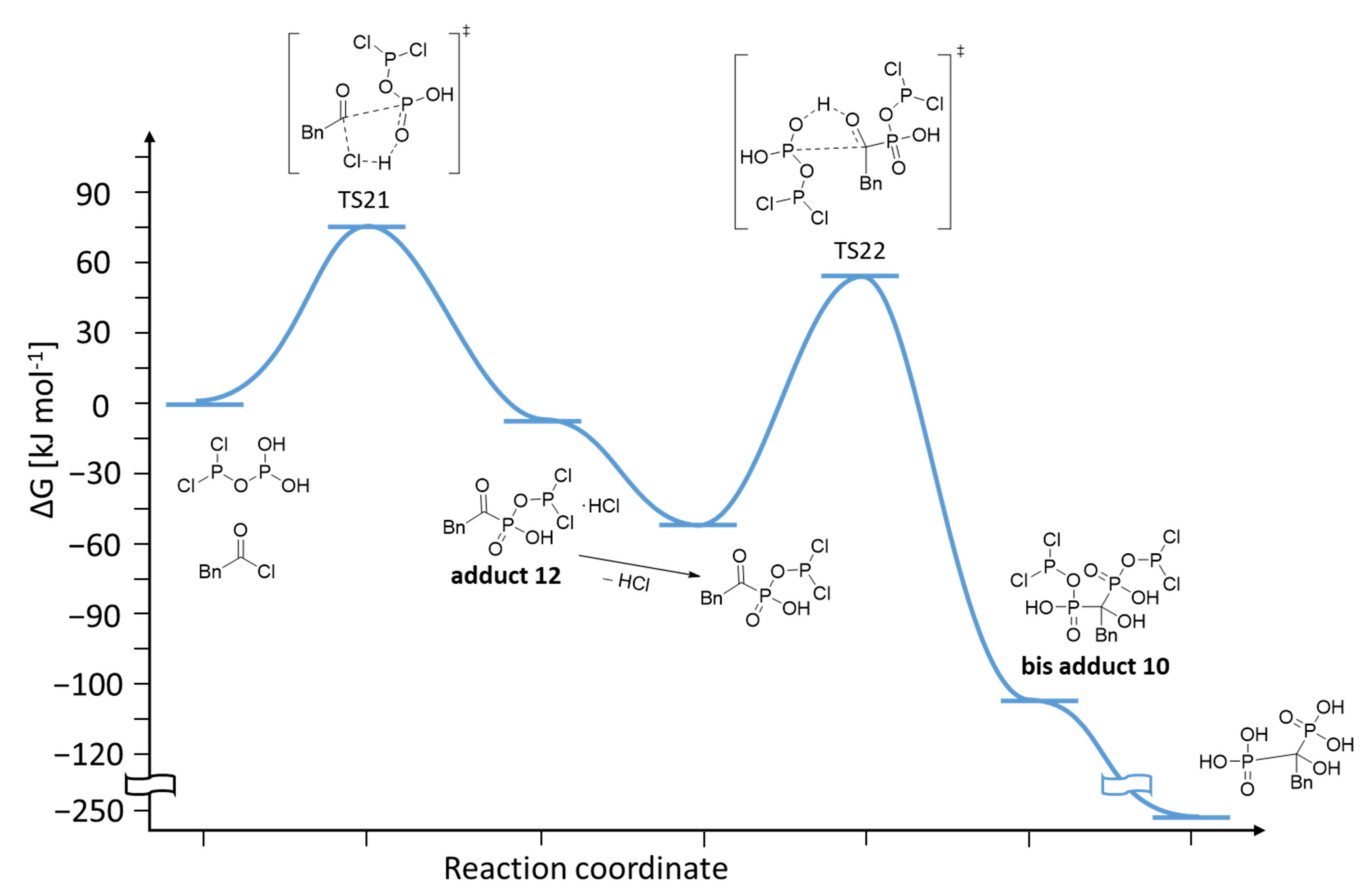
Gibbs free energy diagram for the formation of benzidronic acid from phenylacetyl chloride and Cl_2_P-O-P(OH)_2_.

In the case of the other reaction setup with sulfolane as the solvent, phenylacetyl chloride was reacted with Cl_2_P-O-P(OH)_2_ (Scheme 9). The activation energy required for the first and second additions (**TS21** and **TS22**) were lower for both cases than that for the reaction with benzoyl chloride (**TS13** and **TS14**) (75.0 vs. 137.5 kJ mol^−1^ and 52.8 vs. 94.0 kJ mol^−1^), and both steps were found to be exothermic. The HCl elimination in this case led to an energy gain of −43.9 kJ mol^−1^. The final hydrolysis was again highly exothermic. We have shown the plausible reaction pathway in Figure 5.

The reported yields for benzidronate, in general, were higher than those for fenidronate, which is in accord with the results of the computations suggesting lower activation Gibbs free energies for the formation of benzidronate and steps that are more exothermic before the final hydrolysis. Moreover, in the case of benzidronate, the improving effect of H_3_PO_3_ was shown experimentally, suggesting that the formation of the Cl_2_P-O-P(OH)_2_ reactant is realistic, and results in increased yields. This is also supported by the lower activation energy in the reaction with Cl_2_P-O-P(OH)_2_ as compared to the case with Cl_2_P-O-O_2_SMe as the nucleophile. Notably, in all cases, the acyl chloride was suggested to be the most favorable reactant, implying that the carboxylic acid, in the first step, is transformed by PCl_3_ to the corresponding chloride.

## 3. Conclusions

The possible mechanistic pathways leading to fenidronic acid and benzidronic acid were computed considering the reaction of benzoic acid or phenylacetic acid and derivatives with PCl_3_ in MSA or with PCl_3_/P(OH)_3_ in sulfolane. It was found that the corresponding acyl chlorides should be considered as the starting compounds and PCl_3_ or its condensed derivative with MSA or H_3_PO_4_ (Cl_2_P-O-SO_2_Me or Cl_2_P-O-P(OH)_2_, respectively) may be the nucleophile. The multistep formation of the bis adducts was in most cases endothermic, and the exothermic final hydrolyses could be the driving force for the series of reactions. In the case of benzidronic acid, the activation Gibbs free energies were lower for all steps than that for fenidronic acid. It is noteworthy that in the case of using PCl_3_/P(OH)_3_ in sulfolane, the formation of the adducts was exothermic. It unambiguously turned out that the setup with PCl_3_/P(OH)_3_ in sulfolane involving (HO)_2_*P*-O-PCl_2_ as the nucleophile is the energetically more preferable option. Hence, regarding the synthesis of dronic acid derivatives, the PCl_3_/P(OH)_3_/sulfolane method is recommended.

## 4. Computational Protocol

DFT-level computations at the M062X/6-311+G (d,p) level were performed considering the solvent effect of water (ε = 78.4) or DMSO (ε = 46.8) simulating MSA (no ε is available. However, the physical properties of MSA are similar to that of sulfuric acid (ε_H2SO4_ = 84) [23,24]) and sulfolane (ε = 43.4) [25] on using the SMD solvent model with the Gaussian 09 program package. The actual solvents were chosen based on their similar dielectric constants. The popular M062x method was chosen for its known accuracy for main group thermochemistry and the computation of barrier heights [26]. The geometries of the molecules were optimized in all cases (see Supplementary Material), and frequency calculations were also performed to assure that the structures are in a local minimum or in a saddle point. This was followed by single-point measurements at the M062X/6-311++G (3df,3pd) level, which resulted in the energy values presented in Table S1 and used for the figures of the manuscript. The solution-phase Gibbs free energies were obtained by frequency calculations as well. The G values obtained were given under standard conditions, and the corrected total energies of the molecules were taken into account. Entropic and thermal corrections were evaluated for isolated molecules using standard rigid rotor harmonic oscillator approximations. That is, the Gibbs free energy was taken as the “sum of electronic and thermal free energies” printed in a Gaussian 09 vibrational frequency calculation. Standard state correction was taken into account. The transition states were optimized with the QST3 or the TS (Berny) method. Transition states were identified by having one imaginary frequency in the Hessian matrix and connecting two corresponding minima.

## Figures and Tables

**Table 1:** Preparative results of more important dronic acid derivatives.

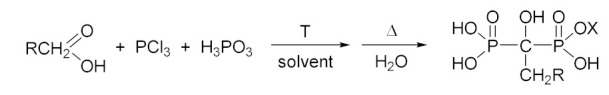								
R	Reagent(s) (equiv.)	Solvent	T (°C)	Workup	Product	Purity (%)	Yield (%)	Ref.
(CH_2_)_2_NH_2_	PCl_3_ (3.2)	MSA	75	pH→1.8 purif.	Pamidronate Na·2H_2_O	99	57	[11]
	PCl_3_/H_3_PO_3_ (2:2)	Sulfolane	75	crystallization	Pamidronic acid	100	63	[11]
(CH_2_)_3_NH_2_	PCl_3_ (3.2)	MSA	75	pH→1.8 purif.	Alendronate Na·3H_2_O	93	58	[12]
	PCl_3_/H_3_PO_3_ (2:2; 3:2)	Sulfolane	75	pH→4.3 crystallization	Alendronate Na·3H_2_O	100/99	46/52	[13]
(CH_2_)_2_NMe^n^Pent	PCl_3_ (3.2)	MSA	75	pH→2 purif.	Ibandronate Na	99	46	[12]
	PCl_3_/H_3_PO_3_ (2:2; 2:3; 2:4)	Sulfolane	75	pH→2 precipitation, digestion (MeOH)	Ibandronate 2Na	100	48/66/73	[14]
CH_2_-2-pyridyl	PCl_3_ (3.1)	MSA	75	pH→2 filtration, washing	Risedronic acid	92	74	[15,16]
	PCl_3_/H_3_PO_3_ (2:2; 2:3; 2:4)	Sulfolane	75	crystallization, digestion (MeOH)	Risedronic acid·H_2_O	100/100/98	58/60/57	[17]
CH_2_-1-imidazolyl	PCl_3_ (3.1)	MSA	80	pH→1.8 recrystallization	Zoledronic acid	98/99	46/53	[15,16]
	PCl_3_/H_3_PO_3_ (2:2; 2:3; 2:4)	Sulfolane	75	pH→1.8 recrystallization	Zoledronic acid·2H_2_O	100/99/99	74/63/66	[18]

## Data Availability

Not applicable.

## Supplementary Materials

The best preparative procedures, together with compound characterization; details of theoretical calculations: Table S1: X, Y, Z Coordinates of the computed structures (columns are in this order), Table S2: Energy (Hartree) and entropy (cal molK–1) values obtained for the computations together with imaginary 2150 frequencies (1/s).

## Sample Availability

Samples of the compounds are available from the authors.
