# Supplementary material for: A Mechanistic Study on the Formation of Dronic Acids
Source: Molecules. 2021 Dec 14;26(24):7587. doi: 10.3390/molecules26247587 (PMC8708247; doi:10.3390/molecules26247587)
Supplement: Supplementary file 1 [file molecules-26-07587-s001.zip › molecules-1497840-supplementary.pdf]

Supplementary Materials:

A Mechanistic Study on the Formation of Dronic Acids

Péter Ábrányi-Balogh [1], István Greiner [2] and György Keglevich [3,*]

[1] Medicinal Chemistry Research Group, Research Centre for Natural Sciences, 1117 Budapest, Hungary; abranyi-balogh.peter@ttk.mta.hu
[2] Gedeon Richter Plc. 1475 Budapest Hungary; i.greiner@richter.hu
[3] Department of Organic Chemistry and Technology, Budapest University of Technology and Economics, 1521 Budapest, Hungary
* Correspondence: keglevich.gyorgy@vbk.bme.hu; Tel.: +36-1-463-1111 (ext. 5883)

1. The Best Synthetic Procedures for the Disodium Salt of Fenidronate and Benzidronate

1.1. Preparation of the Disodium Salt of Fenidronate Starting from Benzoic Acid in a One Step Reaction (Scheme 1/Version A) [S1]

3.1 g (0.025 mol) of benzoic acid was added into 10.5 mL of MSA on stirring. Then 7.0 mL (0.08 mol) of phosphorus trichloride was added dropwise in *ca.* 30 min, and the contents of the flask were stirred at 75 °C for 24 h. After cooling the mixture to 26 °C, 25 mL (1.4 mol) of water was added and the mixture was stirred further at 105 °C for 4 h. The pH was adjusted to 1.8 by adding ~12 mL of 50% aqueous sodium hydroxide to the mixture. Then 127 mL of methanol was added, the mixture stirred for 45 min and the precipitate was removed by filtration. The crude product was dissolved in 10 mL of hot water and 50 mL of methanol was added, then the precipitate was filtered off and dried to give 13.3 g of fenidronate disodium salt (**4**) in a purity of *ca.* 67% (on the basis of ^{31}P NMR). The precipitation was repeated twice by adding 50 mL of methanol to the 10 mL water solution of the crude product. Then the solid product was suspended in the 50 mL mixture of methanol–water 94:6 and the mixture was digested by stirring at 65 °C for 30 min, then the solid product was filtered off. This procedure was repeated twice to afford 3.6 g (46%) of fenidronate disodium salt in a purity of 100%. ^{31}P NMR (D_2O) δ: 16.0, δ[S2] 16.39, ^{13}C NMR (D_2O) δ: 136.9 (t, *J*=2.3, C_1), 128.1 (t, *J*~2.1, C_3*), 127.4 (t, *J*~2.1, C_4), 126.0 (t, *J*=4.4, C_2*), 76.5 (t, *J*=140.9, P–C–P), *may be reversed.

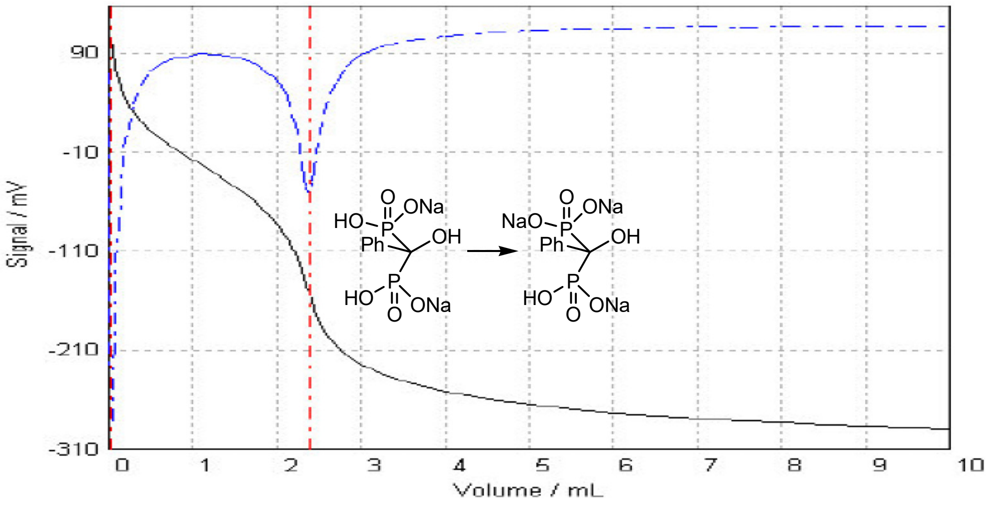

Titration curve for the disodium salt of fenidronic acid obtained by the reaction marked by Scheme 1/*Version A*.

1.2. *Preparation of the Disodium Salt of Benzidronate Starting from Phenylacetic Acid, Phosphorus Trichloride and Phosphorous Acid in a One Step Reaction (Scheme 6/best experiment)* [S3]

3.4 g (0.025 mol) of phenylacetic acid (**5**) and 2.1 g (0.025 mol) phosphorous acid were added into 10.5 mL of MSA on stirring. Then 6.6 mL (0.075 mol) of phosphorus trichloride was added dropwise in *ca.* 30 min, and the contents of the flask were stirred at 85 °C for 24 h. After cooling the mixture to 26 °C, 20 mL (1.1 mol) of water was added and the mixture was stirred further at 105 °C for 4 h. The pH was adjusted to 2.5 by adding ~13 mL of 50% aqueous sodium hydroxide to the mixture. Then, the mixture was stirred for 12 h and the precipitate was removed by filtration to give ~20 g of the crude product that was dissolved in 25 mL of hot water, then 100 mL of methanol was added, and the mixture was stirred for 1 h. The precipitate was filtered off and was suspended in 60 mL of methanol-water 94:6 and the mixture was digested by stirring at 65 °C for 30 min. The solid product was then filtered off to furnish 6.8 g (81%) of benzidronate disodium salt (**6**) in a purity of 98%. ^{31}P NMR (D_2O) δ 17.8, δ[S4] 19.0, ^{13}C NMR (D_2O) δ 38.4 (s, CH_2), 74.3 (t, *J*=134.7, P-C-P), 126.7 (s, C_4), 128.0 (s, C_2*), 131.5 (s, C_3*), 136.8 (t, *J*=7.9, C_1), *may be reversed.

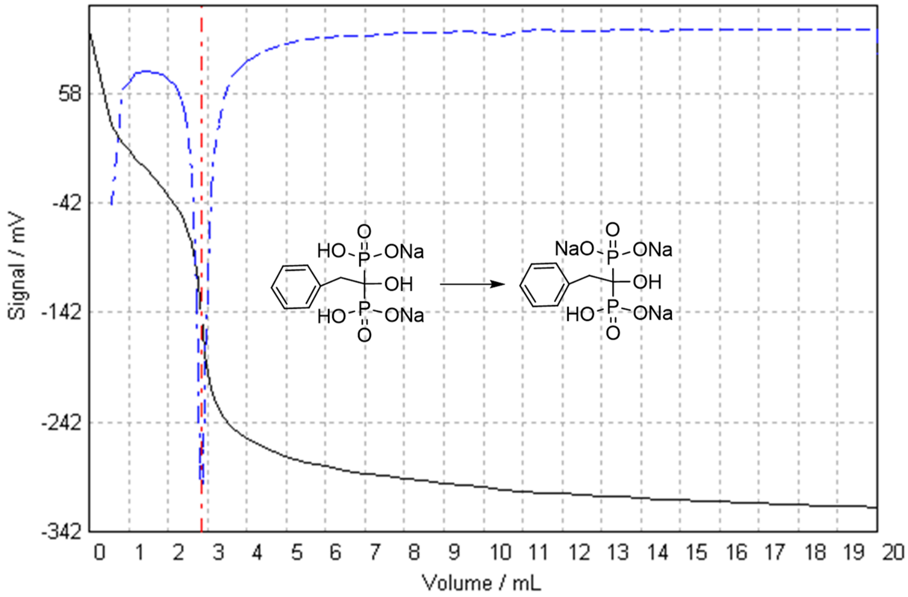

Titration curve for the disodium salt of benzidronic acid obtained by the reaction presented in Scheme 6/best experiment.

References

S1 Grün, A.; Kovács, R.; Nagy, D.I.; Garadnay, S.; Greiner, I.; Keglevich, G. The rational synthesis of Fenidronate. *Lett. Org. Chem.* **2014**, *11*, 368–373. doi:10.2174/1570178611666140124001516

S2 Egorov, M.; Aoun, S.; Padrines, M.; Redini, F.; Heymann, D.; Lebreton, J.; Mathé-Allainmat, M. A one-pot synthesis of 1-hydroxy-1,1-bis(phosphonic acid)s starting from the corresponding carboxylic acids. *Eur. J. Org. Chem.* **2011**, 7148–7154. doi.10.1002/ejoc.201101094

S3 Grün, A.; Kovács, R.; Nagy, D.I.; Garadnay, S.; Greiner, I.; Keglevich, G. Efficient synthesis of benzidronate applying of phosphorus trichloride and phosphorous acid. *Lett. Drug Des. Discov.* **2015**, *12*, 78–84, doi:10.2174/1570180811666141001004732

S4 Agapkina, J.; Yanvarev, D.; Anisenko, A.; Korolev, S.; Vepsäläinen, J.; Kochetkov, S.; Gottikh, M. Specific features of HIV-1 integrase inhibition by bisphosphonate derivatives. *Eur. J. Med. Chem.* **2014**, *73*, 73–82. doi:10.1016/j.ejmech.2013.11.028

2.) Details of Theoretical Calculations

X, Y, Z Coordinates of the computed structures (columns are in this order). Solvents for those species that were used in MSA and in sulfolane as well, are indicated after the name of the molecule.

A1.log

Input orientation:

Center Number	Atomic Number	Atomic Type	Coordinates (Angstroms) X	Y	Z
1	6	0	3.571429	-0.775783	0.865896
2	6	0	2.297430	-1.188388	0.498460
3	6	0	1.547385	-0.403721	-0.380842
4	6	0	2.062012	0.791670	-0.880636
5	6	0	3.342433	1.192763	-0.513166
6	6	0	4.094294	0.413925	0.361148
7	1	0	4.157380	-1.381297	1.546920
8	1	0	1.884776	-2.112537	0.886049
9	1	0	1.471081	1.393961	-1.560370
10	1	0	3.749793	2.115273	-0.909169
11	1	0	5.088804	0.732315	0.650814
12	6	0	0.176164	-0.842650	-0.755111
13	8	0	-0.554820	0.020977	-1.644030
14	8	0	0.116064	-2.176687	-1.015704
15	1	0	-0.778522	-2.460579	-1.255358
16	15	0	-1.218494	0.078597	-0.077321
17	17	0	-3.126814	-0.343563	-0.605546
18	17	0	-1.491794	-0.399046	2.039403
19	17	0	-1.048023	2.075120	0.207573

A10.log

Input orientation:

Center Number	Atomic Number	Atomic Type	Coordinates (Angstroms) X	Y	Z
1	6	0	-3.122779	-1.043078	-1.335595
2	6	0	-2.161270	-0.077390	-1.610967
3	6	0	-1.858808	0.902728	-0.663669
4	6	0	-2.555421	0.926500	0.547232
5	6	0	-3.512691	-0.042585	0.824759
6	6	0	-3.790808	-1.033137	-0.113689
7	1	0	-3.350126	-1.803023	-2.073872
8	1	0	-1.638674	-0.082526	-2.560956
9	1	0	-2.343798	1.706459	1.270671
10	1	0	-4.043185	-0.021943	1.769428
11	1	0	-4.535280	-1.790909	0.101899
12	6	0	0.514157	1.670812	-0.249213
13	8	0	1.447355	2.367236	-0.095379
14	17	0	1.183033	-0.762300	-1.617311
15	6	0	-0.800345	1.934720	-0.937505

16	1	0	-0.557168	2.001383	-2.003058
17	1	0	-1.094346	2.931852	-0.596450
18	15	0	0.772243	-0.155508	0.500463
19	17	0	-0.285667	-1.859636	0.844724
20	17	0	0.225773	0.676778	2.485171
21	17	0	2.710252	-0.421576	1.042507

A10+H.log

Input orientation:

Center Number	Atomic Number	Atomic Type	Coordinates (Angstroms) X	Y	Z
1	6	0	-3.243033	1.178666	-0.388990
2	6	0	-2.241668	1.239668	0.572991
3	6	0	-1.707629	0.063403	1.102166
4	6	0	-2.222442	-1.172210	0.706342
5	6	0	-3.223738	-1.231132	-0.256255
6	6	0	-3.724699	-0.057211	-0.813552
7	1	0	-3.649319	2.094131	-0.802259
8	1	0	-1.870619	2.200133	0.913753
9	1	0	-1.834499	-2.084511	1.146354
10	1	0	-3.614185	-2.192056	-0.569400
11	1	0	-4.502411	-0.105523	-1.567033
12	6	0	0.781245	0.132885	1.562188
13	8	0	1.725881	0.224685	2.376500
14	17	0	0.911946	2.133345	-0.256581
15	6	0	-0.594903	0.132705	2.113084
16	1	0	-0.645763	1.036993	2.735116
17	1	0	-0.609604	-0.716925	2.811300
18	15	0	1.081920	-0.017219	-0.297306
19	17	0	-0.147995	-0.177413	-1.890683
20	17	0	0.988699	-2.138735	0.108392
21	17	0	3.017801	-0.035559	-0.835576
22	1	0	2.654095	0.220055	2.057542

A11.log

Input orientation:

Center Number	Atomic Number	Atomic Type	Coordinates (Angstroms) X	Y	Z
1	6	0	3.900928	-0.053812	1.261611
2	6	0	3.134937	0.753577	0.422140
3	6	0	2.772299	0.299045	-0.843819
4	6	0	3.194851	-0.960247	-1.275312
5	6	0	3.967221	-1.759426	-0.443470
6	6	0	4.314195	-1.309579	0.830608
7	1	0	4.177434	0.302598	2.246935
8	1	0	2.822026	1.740766	0.749721
9	1	0	2.907298	-1.310607	-2.261429

10	1	0	4.295328	-2.734637	-0.783598
11	1	0	4.913127	-1.936354	1.481204
12	6	0	0.453721	0.672690	-1.622860
13	8	0	-0.327185	0.366789	-2.450315
14	17	0	-0.537510	2.761279	0.082160
15	6	0	1.880716	1.127691	-1.736059
16	1	0	1.905697	2.183109	-1.451287
17	1	0	2.142402	1.032317	-2.791369
18	15	0	-0.342813	0.543847	0.180981
19	17	0	0.145885	0.643326	2.163625
20	17	0	0.063653	-1.619779	0.144727
21	8	0	-1.939500	0.438031	0.016669
22	16	0	-3.151729	-0.708391	-0.134424
23	8	0	-2.771746	-1.634582	-1.168292
24	8	0	-4.310802	0.125849	-0.321804
25	6	0	-3.153424	-1.435707	1.471526
26	1	0	-3.248442	-0.627218	2.196057
27	1	0	-4.026524	-2.090269	1.498773
28	1	0	-2.234195	-2.004669	1.597659

A11+H.log

Input orientation:

Center Number	Atomic Number	Atomic Type	Coordinates (Angstroms) X	Y	Z
1	6	0	-4.001677	0.456248	-1.194119
2	6	0	-3.243589	1.011451	-0.169916
3	6	0	-2.726043	0.196698	0.838949
4	6	0	-3.015620	-1.168108	0.843391
5	6	0	-3.775592	-1.721843	-0.181253
6	6	0	-4.256720	-0.912784	-1.207507
7	1	0	-4.393807	1.091649	-1.979468
8	1	0	-3.048282	2.078020	-0.151940
9	1	0	-2.644038	-1.796194	1.645703
10	1	0	-3.991626	-2.783509	-0.178808
11	1	0	-4.842664	-1.346169	-2.009635
12	6	0	-0.416555	0.894683	1.571790
13	8	0	0.331173	1.498210	2.373869
14	17	0	-0.096663	2.103058	-0.813098
15	6	0	-1.854543	0.795743	1.911610
16	1	0	-2.157852	1.814449	2.184199
17	1	0	-1.879801	0.211348	2.842870
18	15	0	0.303782	0.147103	0.010956
19	17	0	-0.541447	-0.794318	-1.561720
20	17	0	0.372943	-1.667962	1.183889
21	8	0	1.863571	0.382691	-0.098473
22	16	0	3.341129	-0.517511	-0.011124
23	8	0	3.423363	-1.046399	1.319311
24	8	0	4.273660	0.472516	-0.464518
25	6	0	3.041711	-1.752827	-1.230469
26	1	0	2.772393	-1.245463	-2.156680
27	1	0	3.987976	-2.287857	-1.340174

28	1	0	2.265355	-2.424134	-0.864111
29	1	0	1.288856	1.582181	2.168934

A12.log

Input orientation:

Center Number	Atomic Number	Atomic Type	Coordinates (Angstroms) X	Y	Z
1	6	0	4.558777	-0.887043	1.104730
2	6	0	3.212205	-0.677764	0.828853
3	6	0	2.811804	-0.256022	-0.440495
4	6	0	3.771349	-0.041013	-1.427321
5	6	0	5.120684	-0.251509	-1.151089
6	6	0	5.515996	-0.675040	0.113964
7	1	0	4.861679	-1.217890	2.091718
8	1	0	2.461996	-0.841787	1.597106
9	1	0	3.462198	0.287056	-2.414408
10	1	0	5.861236	-0.086621	-1.925568
11	1	0	6.565605	-0.841490	0.328323
12	6	0	0.833997	1.229059	-0.088854
13	8	0	1.436873	2.096599	0.466337
14	17	0	-0.413278	-0.536238	2.406355
15	15	0	-2.818737	-0.774097	-1.325482
16	17	0	-3.910827	-0.595074	0.422797
17	17	0	-1.627269	-2.395195	-0.837382
18	8	0	-1.684778	0.429805	-1.071048
19	15	0	-1.029093	1.551832	-0.171984
20	8	0	-1.319893	2.940011	-0.839600
21	1	0	-1.028273	3.067905	-1.760179
22	8	0	-1.648602	1.665018	1.224065
23	1	0	-1.285279	0.864261	1.838388
24	6	0	1.351335	-0.027441	-0.720658
25	1	0	0.737957	-0.852864	-0.342674
26	1	0	1.147276	0.052542	-1.796230

A2.log

Input orientation:

Center Number	Atomic Number	Atomic Type	Coordinates (Angstroms) X	Y	Z
1	6	0	-4.544992	0.166405	-0.765176
2	6	0	-3.218796	0.144299	-1.177371
3	6	0	-2.262581	-0.495632	-0.385710
4	6	0	-2.626688	-1.099955	0.815953
5	6	0	-3.959209	-1.080795	1.216594
6	6	0	-4.916110	-0.446673	0.430457
7	1	0	-5.289812	0.662322	-1.376010
8	1	0	-2.922814	0.618804	-2.105805
9	1	0	-1.876877	-1.590284	1.425397

10	1	0	-4.247221	-1.560736	2.144255
11	1	0	-5.952034	-0.428624	0.748424
12	6	0	-0.840227	-0.501483	-0.828186
13	8	0	0.098421	-1.160404	0.041513
14	8	0	-0.720527	-0.770710	-2.161394
15	1	0	0.206662	-0.802263	-2.442352
16	15	0	0.341088	0.529721	0.045984
17	17	0	0.205548	2.589759	-0.566829
18	17	0	0.078512	0.853065	2.021468
19	8	0	1.921088	0.514919	-0.318371
20	16	0	2.995175	-0.737505	-0.028322
21	8	0	2.733408	-1.217202	1.308773
22	8	0	2.896492	-1.645665	-1.144311
23	6	0	4.462916	0.227238	-0.107768
24	1	0	5.284752	-0.468696	0.068978
25	1	0	4.524038	0.663259	-1.103597
26	1	0	4.408227	0.984551	0.672798

A3.log

Input orientation:

Center Number	Atomic Number	Atomic Type	Coordinates (Angstroms) X	Y	Z
1	6	0	-4.124996	-0.719430	-0.041942
2	6	0	-2.857162	-1.273697	-0.107652
3	6	0	-1.739834	-0.431578	-0.045092
4	6	0	-1.887725	0.949368	0.084009
5	6	0	-3.161399	1.494489	0.146857
6	6	0	-4.275505	0.660954	0.084486
7	1	0	-4.996372	-1.360876	-0.086731
8	1	0	-2.717824	-2.343548	-0.205384
9	1	0	-1.035460	1.618054	0.131183
10	1	0	-3.284132	2.565688	0.243676
11	1	0	-5.269306	1.090167	0.134766
12	6	0	-0.427144	-1.072280	-0.124883
13	8	0	-0.144185	-2.213898	-0.262027
14	17	0	0.955063	0.379441	-2.178201
15	15	0	1.184916	0.055585	0.008324
16	17	0	1.038716	-0.243091	2.199995
17	17	0	2.825064	-1.101454	-0.190051
18	17	0	1.741476	2.003985	0.272913

A3+H.log

Input orientation:

Center Number	Atomic Number	Atomic Type	Coordinates (Angstroms) X	Y	Z
1	6	0	1.688827	3.148455	-0.810473
2	6	0	0.990790	2.042902	-1.247942

3	6	0	0.009158	1.467943	-0.408326
4	6	0	-0.251750	2.015539	0.864986
5	6	0	0.464129	3.114078	1.291040
6	6	0	1.427434	3.680297	0.453847
7	1	0	2.435791	3.602703	-1.448441
8	1	0	1.174051	1.623842	-2.228935
9	1	0	-0.973780	1.578912	1.543799
10	1	0	0.280231	3.530463	2.272505
11	1	0	1.983239	4.546634	0.793326
12	6	0	-0.683474	0.322855	-0.882054
13	8	0	-0.268746	-0.263265	-1.930524
14	17	0	-0.867525	-1.455104	1.203419
15	15	0	-2.240009	-0.339371	-0.032815
16	17	0	-3.279039	1.100868	-1.270643
17	17	0	-3.001520	-1.951307	-0.958765
18	17	0	-3.343511	0.202502	1.584513
19	1	0	-0.769657	-1.021019	-2.293625

A4.log

Input orientation:

Center Number	Atomic Number	Atomic Type	Coordinates (Angstroms) X	Y	Z
1	6	0	-4.227450	0.805133	-0.571227
2	6	0	-2.872957	0.638301	-0.322158
3	6	0	-2.405028	-0.600388	0.117613
4	6	0	-3.281394	-1.675860	0.306454
5	6	0	-4.631821	-1.500371	0.051755
6	6	0	-5.102406	-0.262211	-0.384966
7	1	0	-4.598189	1.763853	-0.910721
8	1	0	-2.207606	1.481453	-0.474024
9	1	0	-2.895546	-2.628855	0.647837
10	1	0	-5.319774	-2.324696	0.192342
11	1	0	-6.159700	-0.128380	-0.581825
12	6	0	-0.989868	-0.833909	0.405074
13	8	0	-0.451297	-1.812431	0.810669
14	17	0	-0.146083	1.269056	2.157723
15	15	0	0.310784	0.585761	0.117148
16	17	0	0.439107	-0.167909	-1.966677
17	17	0	0.445041	2.512855	-0.551623
18	8	0	1.746936	0.075798	0.605068
19	16	0	3.057132	-0.834052	0.068994
20	8	0	3.839000	-0.939828	1.272278
21	8	0	2.549393	-2.024977	-0.557270
22	6	0	3.800317	0.283356	-1.071592
23	1	0	4.753171	-0.169184	-1.352711
24	1	0	3.144382	0.385543	-1.934399
25	1	0	3.951674	1.227851	-0.549467

A4+H.log

Input orientation:

Center Number	Atomic Number	Atomic Type	Coordinates (Angstroms) X	Y	Z
1	6	0	2.119344	-3.297033	0.338557
2	6	0	1.002989	-2.487847	0.332954
3	6	0	0.829187	-1.550487	-0.707927
4	6	0	1.792295	-1.442149	-1.738874
5	6	0	2.907708	-2.251050	-1.711572
6	6	0	3.069515	-3.175827	-0.677305
7	1	0	2.258118	-4.019280	1.131959
8	1	0	0.286810	-2.589307	1.137533
9	1	0	1.649251	-0.723918	-2.535724
10	1	0	3.652992	-2.170711	-2.492264
11	1	0	3.948391	-3.809859	-0.661553
12	6	0	-0.307140	-0.701790	-0.775915
13	8	0	-0.418310	0.117800	-1.738579
14	17	0	-0.296522	0.252978	1.855074
15	15	0	-1.693321	-0.668408	0.515664
16	17	0	-2.867423	-1.679265	-1.023047
17	17	0	-2.248878	-2.045571	1.885078
18	8	0	-2.582125	0.656751	0.525736
19	16	0	-3.453112	1.630451	-0.551867
20	8	0	-3.399419	2.917197	0.070944
21	8	0	-2.790352	1.410937	-1.824448
22	6	0	-5.065224	0.931780	-0.469673
23	1	0	-5.714032	1.634177	-0.997282
24	1	0	-5.049105	-0.040958	-0.955229
25	1	0	-5.326325	0.869863	0.587314
26	1	0	-1.257832	0.642657	-1.843752

A5.log

Input orientation:

Center Number	Atomic Number	Atomic Type	Coordinates (Angstroms) X	Y	Z
1	6	0	-3.338686	-1.787711	1.000613
2	6	0	-2.005793	-1.490430	0.752210
3	6	0	-1.680192	-0.631746	-0.299147
4	6	0	-2.672337	-0.071825	-1.098800
5	6	0	-4.004591	-0.389634	-0.853184
6	6	0	-4.337617	-1.240480	0.196093
7	1	0	-3.599323	-2.448547	1.818462
8	1	0	-1.219574	-1.919121	1.365217
9	1	0	-2.404493	0.598271	-1.907206
10	1	0	-4.781190	0.032304	-1.479584
11	1	0	-5.376908	-1.478938	0.389624
12	6	0	-0.247650	-0.320026	-0.556315
13	8	0	0.028532	0.647088	-1.581152
14	8	0	0.445748	-1.526296	-0.710194
15	16	0	2.003979	-1.753369	-0.267106

16	8	0	2.625761	-0.440950	-0.261508
17	8	0	2.492621	-2.768657	-1.166251
18	6	0	1.823688	-2.383343	1.368015
19	1	0	2.831906	-2.538753	1.755334
20	1	0	1.285498	-1.639989	1.956021
21	1	0	1.274728	-3.322071	1.301430
22	15	0	0.457169	1.281022	-0.089944
23	17	0	-1.064938	2.587737	0.208596
24	17	0	1.146223	1.235513	1.941717
25	17	0	1.986589	2.434151	-0.784003

A6.log

Input orientation:

Center Number	Atomic Number	Atomic Type	Coordinates (Angstroms) X	Y	Z
1	6	0	4.570371	-0.329171	0.166991
2	6	0	3.296226	0.220155	0.200334
3	6	0	2.258486	-0.413460	-0.485903
4	6	0	2.483371	-1.587845	-1.197387
5	6	0	3.767298	-2.123254	-1.237501
6	6	0	4.806479	-1.499177	-0.554192
7	1	0	5.380056	0.155161	0.699480
8	1	0	3.099960	1.134215	0.751894
9	1	0	1.665564	-2.073117	-1.717230
10	1	0	3.953269	-3.031206	-1.798514
11	1	0	5.803218	-1.923496	-0.582673
12	6	0	0.895805	0.185748	-0.429458
13	8	0	-0.162220	-0.549564	-1.061404
14	8	0	0.988497	1.523051	-0.836059
15	16	0	-0.005471	2.711842	-0.313927
16	8	0	-1.209744	2.058996	0.168589
17	8	0	-0.088566	3.630010	-1.422556
18	6	0	0.925822	3.404980	1.012399
19	1	0	0.327038	4.220967	1.420705
20	1	0	1.085045	2.625024	1.756240
21	1	0	1.865199	3.770805	0.599160
22	15	0	-0.387960	-0.545329	0.607244
23	17	0	0.111276	-2.452598	1.056348
24	17	0	-0.304424	0.239418	2.577289
25	8	0	-2.012400	-0.492897	0.628309
26	16	0	-3.081917	-1.018257	-0.539364
27	8	0	-2.502797	-2.186683	-1.159776
28	8	0	-4.311829	-1.139950	0.201795
29	6	0	-3.138011	0.356054	-1.637649
30	1	0	-3.890082	0.100279	-2.386333
31	1	0	-3.436594	1.228253	-1.058865
32	1	0	-2.154163	0.471884	-2.088686

A7.log

Input orientation:

Center Number	Atomic Number	Atomic Type	Coordinates (Angstroms) X	Y	Z
1	6	0	-4.442686	0.413779	-0.219493
2	6	0	-3.155867	0.931333	-0.328058
3	6	0	-2.077782	0.061300	-0.476616
4	6	0	-2.280132	-1.319693	-0.501085
5	6	0	-3.569382	-1.827761	-0.383270
6	6	0	-4.650404	-0.962175	-0.242441
7	1	0	-5.282751	1.089949	-0.113660
8	1	0	-2.993178	2.001472	-0.304442
9	1	0	-1.442249	-1.999426	-0.621178
10	1	0	-3.727384	-2.899348	-0.406525
11	1	0	-5.654297	-1.360469	-0.151690
12	6	0	-0.666753	0.599243	-0.512422
13	8	0	0.162999	-0.186157	-1.304056
14	8	0	-0.651201	1.937770	-0.849538
15	1	0	0.207271	2.164144	-1.235296
16	15	0	1.761053	-0.684710	-0.839000
17	17	0	4.020825	-1.459205	0.165413
18	17	0	2.560699	1.300729	-0.945143
19	8	0	1.298428	-0.590861	0.824262
20	15	0	0.095300	0.347886	1.211511
21	8	0	-0.789245	-0.319467	2.307952
22	1	0	-1.359683	-1.061620	2.031035
23	8	0	0.559391	1.638345	1.958153
24	1	0	1.166171	2.243776	1.494441

A8.log

Input orientation:

Center Number	Atomic Number	Atomic Type	Coordinates (Angstroms) X	Y	Z
1	6	0	-4.003213	0.405129	1.099152
2	6	0	-2.658408	0.291435	0.776433
3	6	0	-2.279269	-0.495421	-0.311734
4	6	0	-3.237960	-1.167054	-1.079738
5	6	0	-4.579098	-1.039523	-0.754828
6	6	0	-4.960211	-0.254621	0.332414
7	1	0	-4.302264	1.005048	1.949629
8	1	0	-1.925927	0.792877	1.397262
9	1	0	-2.926441	-1.771218	-1.923336
10	1	0	-5.328702	-1.548966	-1.348176
11	1	0	-6.010010	-0.159390	0.585217
12	6	0	-0.876033	-0.670991	-0.706323
13	8	0	-0.410623	-1.449854	-1.469977
14	17	0	0.540201	-1.079461	1.728455
15	15	0	0.486406	0.555554	-0.059931
16	17	0	0.089349	1.891708	-1.676542
17	17	0	0.519199	2.018211	1.362164

18	8	0	1.914422	0.154235	-0.517606
19	15	0	3.351748	-0.494631	0.306838
20	8	0	3.075256	-2.061014	0.044597
21	1	0	2.453498	-2.431461	0.690341
22	8	0	4.329113	-0.328483	-0.964311
23	1	0	4.836570	0.496432	-0.941269

A9.log

Input orientation:

Center Number	Atomic Number	Atomic Type	Coordinates (Angstroms) X	Y	Z
1	6	0	-3.845541	1.167364	-0.147075
2	6	0	-2.627692	0.650836	-0.572782
3	6	0	-2.201800	-0.596765	-0.106955
4	6	0	-3.000348	-1.321698	0.786516
5	6	0	-4.208145	-0.796654	1.215208
6	6	0	-4.630783	0.448346	0.748400
7	1	0	-4.176879	2.131964	-0.511808
8	1	0	-2.034071	1.237543	-1.263627
9	1	0	-2.660225	-2.287074	1.141878
10	1	0	-4.822076	-1.352811	1.913436
11	1	0	-5.575710	0.858457	1.086372
12	6	0	-0.923360	-1.212740	-0.523503
13	8	0	-0.603097	-2.349133	-0.291885
14	17	0	3.160113	-2.345192	0.848239
15	15	0	0.855562	2.036114	0.492875
16	17	0	2.919221	1.884963	0.280294
17	17	0	0.552171	0.476292	1.863356
18	8	0	0.368600	1.264686	-0.872597
19	15	0	0.401251	-0.251325	-1.463963
20	8	0	-0.048927	-0.045559	-2.966998
21	1	0	-0.882969	0.423871	-3.137025
22	8	0	1.719782	-0.906194	-1.373499
23	1	0	2.494372	-1.710435	-0.097029

BA1.log

Input orientation:

Center Number	Atomic Number	Atomic Type	Coordinates (Angstroms) X	Y	Z
1	6	0	1.353029	-3.757509	-0.270811
2	6	0	1.033718	-2.714301	0.585248
3	6	0	0.448940	-1.553455	0.067454
4	6	0	0.200999	-1.441487	-1.308259
5	6	0	0.521244	-2.493814	-2.150215
6	6	0	1.097088	-3.649941	-1.633646
7	1	0	1.806505	-4.655923	0.128657
8	1	0	1.234857	-2.799763	1.644506

9	1	0	-0.235402	-0.552020	-1.744203
10	1	0	0.321470	-2.405578	-3.210627
11	1	0	1.349211	-4.467126	-2.298980
12	6	0	0.135498	-0.436469	1.024361
13	8	0	0.443153	-0.760948	2.290901
14	17	0	-1.204815	1.755541	-0.483221
15	15	0	-1.766209	0.030909	0.802987
16	17	0	-2.249732	-1.711732	1.974455
17	17	0	-2.625415	1.280531	2.132575
18	17	0	-2.973011	-0.629730	-0.696009
19	15	0	1.463516	0.999902	0.597838
20	17	0	1.915388	1.318999	-1.307330
21	17	0	3.114166	0.215289	1.386470
22	17	0	1.261212	2.772078	1.485380
23	1	0	0.117667	-0.159488	2.986832

BA10.log

Input orientation:

Center Number	Atomic Number	Atomic Type	Coordinates (Angstroms) X	Y	Z
1	6	0	3.093425	2.871405	-1.497100
2	6	0	1.830548	2.288646	-1.471118
3	6	0	1.338297	1.718622	-0.294217
4	6	0	2.124973	1.761862	0.856337
5	6	0	3.384818	2.353528	0.834465
6	6	0	3.874017	2.906627	-0.343921
7	1	0	3.464788	3.307861	-2.417888
8	1	0	1.219364	2.282981	-2.365701
9	1	0	1.745831	1.330587	1.776914
10	1	0	3.983667	2.377608	1.738291
11	1	0	4.855282	3.367698	-0.364724
12	6	0	-0.308748	-0.144835	-1.033087
13	8	0	0.173872	0.005658	-2.348086
14	15	0	-3.772571	0.532325	1.273966
15	17	0	-5.475358	0.106720	0.139181
16	17	0	-3.448938	2.509987	0.691655
17	8	0	-2.639816	-0.223006	0.370902
18	15	0	-2.146122	-0.337384	-1.179478
19	8	0	-2.289572	-1.905844	-1.458767
20	1	0	-3.064818	-2.182311	-1.975251
21	8	0	-2.830764	0.567651	-2.109797
22	6	0	-0.061252	1.151680	-0.230461
23	1	0	-0.750301	1.903756	-0.632722
24	1	0	-0.344605	0.979986	0.810649
25	15	0	3.237166	-1.846424	0.452843
26	17	0	3.953653	-0.920881	2.156649
27	17	0	3.983832	-0.578622	-1.013106
28	8	0	1.695735	-1.216000	0.560752
29	15	0	0.457957	-1.691846	-0.350802
30	8	0	0.849873	-2.586539	-1.459288
31	1	0	0.366350	-0.866275	-2.726687

32	8	0	-0.487823	-2.281172	0.780267
33	1	0	-1.248815	-2.785151	0.449475

BA2.log

Input orientation:

Center Number	Atomic Number	Atomic Type	Coordinates (Angstroms) X	Y	Z
1	6	0	3.517445	-2.244323	-1.484155
2	6	0	2.338279	-1.516427	-1.557320
3	6	0	1.789169	-0.983869	-0.388925
4	6	0	2.421710	-1.176250	0.844842
5	6	0	3.600380	-1.905871	0.903989
6	6	0	4.148305	-2.439827	-0.258581
7	1	0	3.945407	-2.656327	-2.389653
8	1	0	1.849147	-1.360611	-2.509461
9	1	0	2.020741	-0.759288	1.762937
10	1	0	4.087911	-2.053079	1.859432
11	1	0	5.068599	-3.009422	-0.209193
12	6	0	0.501997	-0.206965	-0.485458
13	8	0	0.037099	-0.177525	-1.763044
14	17	0	-0.108290	1.242666	2.078026
15	15	0	0.885754	1.640632	0.141544
16	17	0	1.914545	1.977670	-1.731158
17	17	0	-0.388404	3.184346	-0.206868
18	17	0	2.580381	2.127543	1.163640
19	1	0	-0.784933	0.329865	-1.904679
20	6	0	-4.265338	1.129768	0.459412
21	1	0	-5.101198	1.525350	-0.123511
22	1	0	-4.610279	0.589037	1.340185
23	1	0	-3.532781	1.902092	0.692555
24	16	0	-3.478189	-0.033093	-0.593741
25	8	0	-2.212938	-0.461393	0.544173
26	8	0	-4.228394	-1.232173	-0.804481
27	8	0	-2.742697	0.575911	-1.673480
28	15	0	-0.851228	-1.216032	0.445074
29	17	0	-0.489001	-2.007476	2.217605
30	17	0	-1.081377	-2.775984	-0.771142

BA3.log

Input orientation:

Center Number	Atomic Number	Atomic Type	Coordinates (Angstroms) X	Y	Z
1	6	0	-3.739625	1.844633	0.694026
2	6	0	-2.501055	1.220761	0.705728
3	6	0	-1.908585	0.833689	-0.503736
4	6	0	-2.574972	1.060101	-1.714442
5	6	0	-3.814661	1.682439	-1.709820

6	6	0	-4.398295	2.075620	-0.510052
7	1	0	-4.189702	2.147265	1.631046
8	1	0	-2.025173	1.044914	1.662243
9	1	0	-2.124576	0.759355	-2.650160
10	1	0	-4.322792	1.858307	-2.649879
11	1	0	-5.366584	2.561649	-0.512202
12	6	0	-0.573013	0.134951	-0.573112
13	8	0	-0.198587	-0.042657	-1.852614
14	17	0	0.259654	-0.096674	2.295163
15	15	0	0.780770	1.064096	0.499845
16	17	0	1.275761	2.275398	-1.209558
17	17	0	0.419361	2.761447	1.563957
18	8	0	2.238512	0.391368	0.612078
19	16	0	3.333730	-0.308787	-0.466061
20	8	0	3.915544	-1.359128	0.317308
21	8	0	2.541291	-0.639028	-1.632839
22	6	0	4.475500	0.999160	-0.746469
23	1	0	5.296056	0.547476	-1.308673
24	1	0	3.978590	1.776779	-1.321677
25	1	0	4.810821	1.349946	0.229403
26	15	0	-0.939638	-1.729544	0.048624
27	17	0	-1.768325	-2.526803	-1.575401
28	17	0	-2.294304	-1.988149	1.475514
29	17	0	0.639569	-2.873885	0.465790
30	1	0	0.731537	-0.310106	-1.993758

BA4.log

Input orientation:

Center Number	Atomic Number	Atomic Type	Coordinates (Angstroms) X	Y	Z
1	6	0	4.331510	-1.116621	0.636125
2	6	0	3.003656	-0.718942	0.702156
3	6	0	2.301557	-0.460903	-0.480102
4	6	0	2.928566	-0.590942	-1.720223
5	6	0	4.257704	-0.987779	-1.772495
6	6	0	4.958293	-1.252289	-0.599252
7	1	0	4.872244	-1.322181	1.551387
8	1	0	2.539028	-0.612707	1.677664
9	1	0	2.380525	-0.392519	-2.631755
10	1	0	4.744163	-1.094098	-2.734344
11	1	0	5.994187	-1.566323	-0.645992
12	6	0	0.849131	-0.051734	-0.443157
13	8	0	0.328003	0.027355	-1.699369
14	17	0	-0.358031	0.036508	2.285594
15	15	0	-0.163073	-1.365099	0.597409
16	17	0	0.160738	-2.764579	-1.014335
17	17	0	0.553456	-2.755711	1.910079
18	8	0	-1.746773	-1.259960	0.430812
19	16	0	-2.976305	-1.654459	-0.684779
20	8	0	-4.079585	-0.900359	-0.159073
21	8	0	-2.454368	-1.364196	-1.994050

22	6	0	-3.177517	-3.376081	-0.374121
23	1	0	-4.074114	-3.668962	-0.924356
24	1	0	-2.299432	-3.900438	-0.744350
25	1	0	-3.317716	-3.496883	0.699899
26	6	0	-2.274460	4.053341	-0.665233
27	1	0	-3.314639	4.147068	-0.986316
28	1	0	-2.034722	4.762752	0.125995
29	1	0	-1.589057	4.115152	-1.509951
30	16	0	-2.136756	2.441113	0.017857
31	8	0	-0.472272	2.541457	0.512240
32	8	0	-2.851674	2.285216	1.244323
33	8	0	-2.126562	1.397422	-0.978712
34	15	0	0.866592	1.800080	0.180625
35	17	0	1.649686	2.718373	-1.405840
36	17	0	2.077200	2.245541	1.680284
37	1	0	-0.626402	0.225367	-1.733776

BA5.log

Input orientation:

Center Number	Atomic Number	Atomic Type	Coordinates (Angstroms) X	Y	Z
1	6	0	0.634136	2.772896	0.717197
2	6	0	0.280951	1.431665	0.619934
3	6	0	0.050376	0.853146	-0.632773
4	6	0	0.198295	1.627625	-1.782263
5	6	0	0.559525	2.967929	-1.679742
6	6	0	0.775317	3.544392	-0.433145
7	1	0	0.804112	3.209880	1.694460
8	1	0	0.191513	0.845537	1.528188
9	1	0	0.021670	1.201936	-2.762451
10	1	0	0.667898	3.559674	-2.581174
11	1	0	1.053010	4.589165	-0.356722
12	6	0	-0.282242	-0.629184	-0.778995
13	8	0	-0.848694	-0.957956	-2.022686
14	15	0	-3.513523	1.039988	0.750638
15	17	0	-5.106700	-0.291994	0.944468
16	17	0	-3.367195	1.088468	-1.329578
17	8	0	-2.259699	0.041932	1.084825
18	15	0	-1.505560	-1.258401	0.468733
19	8	0	-0.501790	-1.594860	1.671596
20	1	0	-0.906146	-1.779887	2.536735
21	8	0	-2.402506	-2.324616	0.016429
22	17	0	5.030678	-0.630762	0.005908
23	17	0	3.383179	0.843779	2.279413
24	15	0	3.253187	0.431445	0.253091
25	15	0	1.332242	-1.577184	-0.712427
26	8	0	2.233717	-0.875517	0.426982
27	8	0	1.105329	-3.037744	-0.146429
28	1	0	0.874538	-3.096944	0.794893
29	8	0	1.914419	-1.554399	-2.066316
30	1	0	-0.143977	-0.984190	-2.688658

BA6.log

Input orientation:

Center Number	Atomic Number	Atomic Type	Coordinates (Angstroms) X	Y	Z
1	6	0	-3.948959	-1.748188	1.111412
2	6	0	-2.560171	-1.794221	1.074076
3	6	0	-1.880142	-1.590769	-0.127964
4	6	0	-2.603881	-1.360280	-1.299339
5	6	0	-3.992964	-1.305463	-1.257829
6	6	0	-4.666785	-1.493464	-0.053623
7	1	0	-4.468266	-1.904329	2.049489
8	1	0	-1.997879	-1.977916	1.984103
9	1	0	-2.086376	-1.226545	-2.241742
10	1	0	-4.549741	-1.120387	-2.168809
11	1	0	-5.749290	-1.449556	-0.025713
12	6	0	0.292508	-0.381797	-0.661944
13	8	0	0.217597	-0.356216	-1.991509
14	17	0	0.551419	0.484241	2.076035
15	6	0	-0.367516	-1.657073	-0.117853
16	1	0	-0.042785	-1.868727	0.897557
17	1	0	-0.015706	-2.464036	-0.771338
18	15	0	-0.446247	1.240200	0.238770
19	17	0	-2.161710	1.457380	1.315395
20	17	0	-1.482129	1.854476	-1.549296
21	17	0	0.676814	2.929343	0.260868
22	15	0	2.258002	-0.612099	-0.237193
23	17	0	3.380130	0.957150	0.230839
24	17	0	3.014225	-1.294771	-1.944518
25	17	0	2.631766	-2.015660	1.116447
26	1	0	0.487355	0.480850	-2.410792

BA7.log

Input orientation:

Center Number	Atomic Number	Atomic Type	Coordinates (Angstroms) X	Y	Z
1	6	0	-3.429124	2.646030	-0.903210
2	6	0	-2.123856	2.278344	-0.597469
3	6	0	-1.807953	1.770079	0.664479
4	6	0	-2.809512	1.697388	1.636546
5	6	0	-4.112872	2.073903	1.334763
6	6	0	-4.428208	2.534289	0.059297
7	1	0	-3.661148	3.026779	-1.890821
8	1	0	-1.354784	2.401462	-1.349746
9	1	0	-2.565338	1.340474	2.630666
10	1	0	-4.881225	2.005730	2.095799
11	1	0	-5.446390	2.819103	-0.179373

12	6	0	0.064349	-0.024064	0.423493
13	8	0	0.821958	-0.666996	1.369721
14	17	0	-1.204061	-0.516608	-2.210816
15	6	0	-0.405702	1.330491	1.020010
16	1	0	0.320215	2.110192	0.766412
17	1	0	-0.324394	1.177707	2.098084
18	15	0	-1.471276	-1.134888	-0.119124
19	17	0	-3.492532	-0.970651	-0.418300
20	17	0	-1.836896	-1.510597	1.974795
21	17	0	-0.813863	-3.017144	-0.488417
22	1	0	1.085568	-1.581223	1.159473
23	15	0	1.408775	0.338776	-0.897980
24	17	0	2.054120	-1.316724	-1.775078
25	17	0	1.134879	1.738152	-2.265907
26	8	0	2.567914	0.941541	-0.044362
27	16	0	3.717425	0.360554	1.144609
28	8	0	3.316842	1.024280	2.346972
29	8	0	3.688457	-1.070378	1.029739
30	6	0	5.133201	1.081958	0.391984
31	1	0	5.240658	0.631612	-0.594082
32	1	0	4.969749	2.157787	0.347890
33	1	0	5.971874	0.829994	1.044653

BA8.log

Input orientation:

Center Number	Atomic Number	Atomic Type	Coordinates (Angstroms) X	Y	Z
1	6	0	4.614273	-0.619640	0.207701
2	6	0	3.611134	-0.010970	0.952043
3	6	0	2.673037	0.818784	0.332126
4	6	0	2.788461	1.069099	-1.036815
5	6	0	3.788916	0.457047	-1.783072
6	6	0	4.696923	-0.396980	-1.163986
7	1	0	5.330321	-1.266889	0.700090
8	1	0	3.548483	-0.182973	2.020662
9	1	0	2.106429	1.748146	-1.532437
10	1	0	3.861758	0.654099	-2.846082
11	1	0	5.475652	-0.875532	-1.746246
12	6	0	0.180269	0.806969	1.083266
13	8	0	-0.359606	0.747707	2.323111
14	17	0	0.985263	-1.740765	2.126053
15	6	0	1.587260	1.454232	1.171447
16	1	0	1.838560	1.372753	2.230894
17	1	0	1.532006	2.526188	0.956936
18	15	0	0.218301	-0.979787	0.260644
19	17	0	1.539842	-2.192764	-0.737864
20	17	0	-0.315733	-0.122667	-1.692931
21	8	0	-1.212977	-1.633432	0.533200
22	16	0	-2.507908	-2.386743	-0.279746
23	8	0	-3.141965	-1.388972	-1.095137
24	8	0	-3.215881	-2.975125	0.822097

25	6	0	-1.658199	-3.595632	-1.238455
26	1	0	-1.027732	-4.175755	-0.564835
27	1	0	-2.441110	-4.220259	-1.674932
28	1	0	-1.097423	-3.087416	-2.022494
29	15	0	-0.980286	2.123683	0.171178
30	17	0	-2.817371	1.559820	-0.304252
31	17	0	-0.224743	3.139079	-1.360278
32	17	0	-1.195408	3.494741	1.601936
33	1	0	-1.181395	0.229278	2.394130

BA9.log

Input orientation:

Center Number	Atomic Number	Atomic Type	Coordinates (Angstroms) X	Y	Z
1	6	0	1.248085	4.565820	0.068832
2	6	0	0.864827	3.580562	0.971294
3	6	0	-0.076237	2.614330	0.607122
4	6	0	-0.657334	2.674960	-0.661553
5	6	0	-0.269708	3.656792	-1.566885
6	6	0	0.689955	4.598271	-1.206283
7	1	0	1.983852	5.305941	0.360739
8	1	0	1.301315	3.554022	1.963452
9	1	0	-1.412562	1.953606	-0.949769
10	1	0	-0.720189	3.686378	-2.551945
11	1	0	0.994186	5.361783	-1.912769
12	6	0	0.041319	0.109685	1.325487
13	8	0	0.387737	-0.472487	2.518578
14	17	0	2.766523	0.885703	1.781069
15	6	0	-0.457878	1.551808	1.612523
16	1	0	-0.021530	1.787520	2.584563
17	1	0	-1.543434	1.552414	1.755260
18	15	0	1.605367	0.115391	0.123366
19	17	0	2.587832	1.432056	-1.113951
20	17	0	0.383555	-0.449897	-1.604521
21	8	0	2.313349	-1.317292	0.270789
22	16	0	2.824132	-2.628152	-0.678054
23	8	0	1.648631	-3.300780	-1.158841
24	8	0	3.712703	-3.298770	0.230532
25	6	0	3.705346	-1.812030	-1.966455
26	1	0	4.449135	-1.162518	-1.505558
27	1	0	4.185602	-2.609342	-2.538310
28	1	0	2.992661	-1.273357	-2.589933
29	15	0	-1.467515	-0.915485	0.758128
30	17	0	-1.010684	-2.787424	0.298816
31	17	0	-2.569340	-1.045964	2.414002
32	1	0	0.813744	-1.343584	2.439965
33	8	0	-2.318377	-0.254328	-0.353346
34	16	0	-4.001056	0.228672	-0.679000
35	8	0	-3.809407	1.096994	-1.797604
36	8	0	-4.487996	0.711515	0.577933
37	6	0	-4.637576	-1.349997	-1.125496

38	1	0	-5.660300	-1.168365	-1.465232
39	1	0	-4.010740	-1.735284	-1.929221
40	1	0	-4.621520	-1.979004	-0.235123

TS1.log

Input orientation:

Center Number	Atomic Number	Atomic Type	Coordinates (Angstroms) X	Y	Z
1	6	0	-3.566268	0.308882	1.176606
2	6	0	-2.337666	0.909455	0.946336
3	6	0	-1.604464	0.554854	-0.190175
4	6	0	-2.091155	-0.395017	-1.089187
5	6	0	-3.328844	-0.982967	-0.853763
6	6	0	-4.061238	-0.636659	0.277865
7	1	0	-4.139262	0.574702	2.056661
8	1	0	-1.942167	1.642524	1.639671
9	1	0	-1.514261	-0.653051	-1.969089
10	1	0	-3.719887	-1.711020	-1.554105
11	1	0	-5.022211	-1.103083	0.461305
12	6	0	-0.294742	1.190378	-0.447442
13	8	0	0.474076	0.771607	-1.502952
14	8	0	-0.232561	2.467794	-0.091206
15	1	0	0.576980	2.886593	-0.426755
16	15	0	1.197359	-0.003248	-0.011666
17	17	0	3.074070	0.637260	-0.477480
18	17	0	1.758155	-0.700679	2.054371
19	17	0	1.024262	-1.889358	-0.764993

TS10.log

Input orientation:

Center Number	Atomic Number	Atomic Type	Coordinates (Angstroms) X	Y	Z
1	6	0	4.228943	-1.375761	0.728028
2	6	0	2.868868	-1.122588	0.697344
3	6	0	2.237791	-0.879707	-0.534454
4	6	0	2.982062	-0.871749	-1.727063
5	6	0	4.341910	-1.122809	-1.678061
6	6	0	4.963894	-1.374297	-0.455793
7	1	0	4.717481	-1.561375	1.675914
8	1	0	2.327204	-1.084868	1.634972
9	1	0	2.489670	-0.684301	-2.672783
10	1	0	4.919434	-1.125294	-2.593812
11	1	0	6.029457	-1.568737	-0.426053
12	6	0	0.812218	-0.570131	-0.612676
13	8	0	0.328832	-0.373470	-1.799983
14	17	0	-0.213179	0.002764	2.176308
15	15	0	-0.363222	-1.509573	0.621742

16	17	0	-0.349858	-3.060773	-0.909129
17	17	0	0.179598	-2.918340	1.988874
18	8	0	-1.919754	-1.185484	0.508521
19	16	0	-3.132717	-1.190656	-0.690123
20	8	0	-4.087709	-0.260582	-0.166531
21	8	0	-2.434441	-0.917594	-1.927087
22	6	0	-3.740254	-2.839072	-0.577514
23	1	0	-4.658391	-2.849004	-1.168707
24	1	0	-2.997886	-3.519099	-0.986972
25	1	0	-3.948398	-3.030136	0.475277
26	6	0	-1.928800	4.226706	-0.499731
27	1	0	-2.987979	4.271210	-0.761372
28	1	0	-1.665993	5.000554	0.220620
29	1	0	-1.300150	4.257881	-1.388691
30	16	0	-1.693528	2.670917	0.280428
31	8	0	-0.058533	2.783596	0.649318
32	8	0	-2.382093	2.595485	1.537664
33	8	0	-1.754314	1.566446	-0.657112
34	15	0	1.016444	1.707221	0.059098
35	17	0	1.554533	2.536231	-1.719230
36	17	0	2.573078	2.111194	1.269607
37	1	0	-0.649805	-0.314699	-1.893512

TS11.log

Input orientation:

Center Number	Atomic Number	Atomic Type	Coordinates (Angstroms) X	Y	Z
1	6	0	-4.446803	0.468607	0.079660
2	6	0	-3.168594	1.005221	-0.028931
3	6	0	-2.133673	0.218864	-0.534828
4	6	0	-2.371385	-1.101161	-0.918334
5	6	0	-3.652034	-1.632533	-0.800992
6	6	0	-4.689087	-0.848604	-0.302869
7	1	0	-5.254257	1.080002	0.465337
8	1	0	-2.976189	2.027469	0.274179
9	1	0	-1.559573	-1.702034	-1.313624
10	1	0	-3.839215	-2.656465	-1.102751
11	1	0	-5.686612	-1.263276	-0.213243
12	6	0	-0.728274	0.750970	-0.614901
13	8	0	0.097622	0.154190	-1.422725
14	8	0	-0.685147	2.116008	-0.560833
15	1	0	0.157940	2.402896	-0.941353
16	15	0	1.966021	-0.547810	-0.898710
17	17	0	3.945596	-1.346383	-0.126048
18	17	0	2.568192	1.488935	-0.603043
19	8	0	1.298910	-0.797130	0.612806
20	15	0	0.070316	0.053481	1.231450
21	8	0	-0.721376	-0.976717	2.137304
22	1	0	-1.336555	-1.558523	1.657902
23	8	0	0.667901	0.966556	2.385295
24	1	0	1.090845	1.790034	2.089401

TS12.log

Input orientation:

Center Number	Atomic Number	Atomic Type	Coordinates (Angstroms) X	Y	Z
1	6	0	3.995680	0.318134	1.143708
2	6	0	2.724155	0.676764	0.716634
3	6	0	2.278507	0.247305	-0.534449
4	6	0	3.097981	-0.535135	-1.353296
5	6	0	4.369497	-0.884728	-0.916069
6	6	0	4.817063	-0.461731	0.331580
7	1	0	4.349622	0.657539	2.109807
8	1	0	2.100132	1.308992	1.334860
9	1	0	2.734315	-0.871768	-2.316824
10	1	0	5.006000	-1.492104	-1.548704
11	1	0	5.808477	-0.737481	0.672022
12	6	0	0.906188	0.481815	-1.060478
13	8	0	0.524353	0.407778	-2.183524
14	17	0	0.262268	2.538899	-0.174033
15	15	0	-0.477542	-0.285514	0.192615
16	17	0	-0.308834	-2.317716	0.019146
17	17	0	-0.419236	-0.001007	2.197540
18	8	0	-1.893661	0.059629	-0.313540
19	15	0	-3.575657	-0.019428	0.261931
20	8	0	-4.114783	1.135637	-0.717819
21	1	0	-4.028041	2.026729	-0.345410
22	8	0	-4.051408	-1.232259	-0.684445
23	1	0	-4.104324	-2.081597	-0.220190

TS13.log

Input orientation:

Center Number	Atomic Number	Atomic Type	Coordinates (Angstroms) X	Y	Z
1	6	0	4.293262	1.036025	0.883798
2	6	0	2.980087	1.046279	0.432821
3	6	0	2.529903	0.026382	-0.405982
4	6	0	3.390483	-0.998086	-0.792671
5	6	0	4.709107	-0.997180	-0.345822
6	6	0	5.160114	0.015739	0.492963
7	1	0	4.644423	1.828761	1.533929
8	1	0	2.319852	1.859643	0.716429
9	1	0	3.023776	-1.786147	-1.439353
10	1	0	5.380719	-1.790714	-0.652296
11	1	0	6.185734	0.014195	0.843690
12	6	0	1.101254	-0.065717	-0.889345
13	8	0	0.711387	-0.953226	-1.639996
14	17	0	0.549013	1.740212	-1.448536

15	15	0	-3.090697	-0.229920	0.779747
16	17	0	-3.241463	-1.481463	-0.881660
17	17	0	-3.984278	1.471772	0.024424
18	8	0	-1.530366	0.294411	0.526020
19	15	0	-0.060348	-0.304919	0.775315
20	8	0	0.291969	-0.021721	2.298626
21	1	0	0.637397	0.867397	2.488652
22	8	0	-0.257349	-1.879300	0.836024
23	1	0	-0.132251	-2.291362	-0.038830

TS14.log

Input orientation:

Center Number	Atomic Number	Atomic Type	Coordinates (Angstroms) X	Y	Z
1	6	0	0.705030	2.568985	0.997857
2	6	0	0.272681	1.262348	0.800998
3	6	0	0.019527	0.800287	-0.494186
4	6	0	0.203278	1.647132	-1.584696
5	6	0	0.638323	2.953860	-1.382288
6	6	0	0.890539	3.415398	-0.094468
7	1	0	0.905398	2.921630	2.002835
8	1	0	0.148774	0.608442	1.659496
9	1	0	0.002642	1.272428	-2.581090
10	1	0	0.781766	3.610689	-2.232390
11	1	0	1.233208	4.431980	0.061018
12	6	0	-0.425580	-0.625666	-0.787926
13	8	0	-0.571069	-0.992040	-2.001741
14	15	0	-3.736487	1.021366	0.475143
15	17	0	-5.370511	-0.279371	0.535269
16	17	0	-3.392917	1.026850	-1.587528
17	8	0	-2.544866	0.013036	0.943677
18	15	0	-1.740619	-1.295673	0.373028
19	8	0	-0.888889	-1.674317	1.686454
20	1	0	-1.411351	-1.922188	2.469095
21	8	0	-2.608345	-2.347183	-0.170246
22	17	0	5.041793	-0.227666	-0.856152
23	17	0	3.927617	1.005337	1.847981
24	15	0	3.277210	0.557128	-0.068822
25	15	0	1.313396	-1.712455	-0.260556
26	8	0	2.570206	-0.896985	0.340687
27	8	0	1.390029	-3.089357	0.525580
28	1	0	0.955143	-3.075379	1.394141
29	8	0	1.676449	-2.078790	-1.743492
30	1	0	0.865367	-1.755985	-2.259956

TS15.log

Input orientation:

Center Atomic Atomic Coordinates (Angstroms)

Number	Number	Type	X	Y	Z
1	6	0	-2.934827	-0.501706	-1.301034
2	6	0	-1.902888	0.434105	-1.289061
3	6	0	-1.717653	1.245426	-0.170269
4	6	0	-2.578847	1.142869	0.922484
5	6	0	-3.617462	0.221987	0.898881
6	6	0	-3.789467	-0.607205	-0.209311
7	1	0	-3.070044	-1.141439	-2.165078
8	1	0	-1.241994	0.534983	-2.144584
9	1	0	-2.428603	1.782291	1.785818
10	1	0	-4.288491	0.144525	1.745971
11	1	0	-4.595928	-1.331058	-0.222249
12	6	0	0.643995	1.681291	0.546202
13	8	0	1.526801	1.909645	1.222679
14	17	0	1.852769	0.671298	-1.912260
15	6	0	-0.573149	2.216450	-0.127825
16	1	0	-0.226193	2.507489	-1.123564
17	1	0	-0.795474	3.113919	0.460598
18	15	0	0.761526	-0.625775	0.089203
19	17	0	0.334372	-2.125109	-1.243620
20	17	0	-0.338691	-1.458368	1.723872
21	17	0	2.637827	-1.115652	0.758454

TS16.log

Input orientation:

Center Number	Atomic Number	Atomic Type	Coordinates (Angstroms) X	Y	Z
1	6	0	3.995503	0.649077	1.498700
2	6	0	2.816610	1.332189	1.214454
3	6	0	2.226314	1.235287	-0.046421
4	6	0	2.842652	0.451463	-1.025343
5	6	0	4.015070	-0.240425	-0.738164
6	6	0	4.594019	-0.145046	0.524975
7	1	0	4.444480	0.737803	2.481294
8	1	0	2.349774	1.946794	1.977535
9	1	0	2.407174	0.382678	-2.015236
10	1	0	4.481699	-0.846491	-1.506316
11	1	0	5.509267	-0.681529	0.747399
12	6	0	-0.143345	1.137445	-0.886518
13	8	0	-0.057446	0.362091	-1.805507
14	17	0	-1.660300	2.629838	-1.322196
15	6	0	0.969630	2.024492	-0.351574
16	1	0	0.625708	2.576661	0.525055
17	1	0	1.158131	2.742096	-1.154212
18	15	0	-1.193979	0.271103	0.536770
19	17	0	-2.832825	1.016664	1.402504
20	17	0	-0.028310	-0.252987	2.094078
21	8	0	-1.739338	-1.073780	-0.110841
22	16	0	-0.923940	-2.397417	-0.821241
23	8	0	0.464607	-2.242250	-0.469168

24	8	0	-1.345457	-2.402640	-2.192376
25	6	0	-1.715247	-3.657295	0.117760
26	1	0	-2.785866	-3.582504	-0.066570
27	1	0	-1.313438	-4.602543	-0.251542
28	1	0	-1.462828	-3.495876	1.164800

TS17.log

Input orientation:

Center Number	Atomic Number	Atomic Type	Coordinates (Angstroms) X	Y	Z
1	6	0	-3.918184	-1.759138	1.135790
2	6	0	-2.541406	-1.893224	1.017703
3	6	0	-1.921763	-1.652439	-0.211083
4	6	0	-2.688463	-1.280893	-1.319977
5	6	0	-4.065456	-1.136744	-1.192611
6	6	0	-4.679004	-1.367952	0.035200
7	1	0	-4.397700	-1.952047	2.088110
8	1	0	-1.942805	-2.186759	1.872968
9	1	0	-2.215174	-1.118571	-2.283166
10	1	0	-4.656926	-0.847711	-2.053080
11	1	0	-5.751973	-1.251847	0.134326
12	6	0	0.093343	-0.432912	-0.780143
13	8	0	0.321076	-0.349894	-2.038221
14	17	0	-0.045804	0.247145	2.039321
15	6	0	-0.413866	-1.779989	-0.328950
16	1	0	0.007364	-2.097056	0.623256
17	1	0	-0.126184	-2.488502	-1.109264
18	15	0	-0.622382	1.160694	0.164727
19	17	0	-2.474828	1.543405	0.922909
20	17	0	-1.283540	1.974451	-1.749250
21	17	0	0.698022	2.680116	0.422333
22	15	0	2.376797	-0.535878	-0.027327
23	17	0	3.604565	0.965120	-0.597993
24	17	0	2.950912	-2.069770	-1.215719
25	17	0	3.055949	-1.074443	1.791345
26	1	0	0.481528	0.547937	-2.404353

TS18.log

Input orientation:

Center Number	Atomic Number	Atomic Type	Coordinates (Angstroms) X	Y	Z
1	6	0	-2.722878	2.894339	-1.139178
2	6	0	-1.637044	2.295219	-0.511466
3	6	0	-1.759830	1.795048	0.787109
4	6	0	-2.965974	1.959201	1.472393
5	6	0	-4.047532	2.570157	0.849646
6	6	0	-3.932127	3.023784	-0.462112

7	1	0	-2.620324	3.267803	-2.150942
8	1	0	-0.689490	2.226065	-1.034173
9	1	0	-3.056046	1.603689	2.492647
10	1	0	-4.981146	2.687797	1.386578
11	1	0	-4.779161	3.491173	-0.950560
12	6	0	-0.394286	-0.328883	1.028437
13	8	0	0.284259	-1.060015	1.820764
14	17	0	-0.984294	-0.763332	-1.956339
15	6	0	-0.609728	1.096837	1.467991
16	1	0	0.337132	1.633334	1.345021
17	1	0	-0.773220	1.020805	2.549353
18	15	0	-1.841605	-1.178344	-0.022933
19	17	0	-3.664403	-0.597656	-0.727588
20	17	0	-2.703607	-1.350502	1.985081
21	17	0	-1.560328	-3.172139	-0.186072
22	1	0	0.416451	-2.007657	1.603537
23	15	0	1.577389	-0.003074	-0.558642
24	17	0	2.458878	-1.695380	-1.284184
25	17	0	1.846716	1.308476	-2.100455
26	8	0	2.594656	0.536189	0.567702
27	16	0	4.228482	0.478470	0.962253
28	8	0	4.291234	1.299727	2.140281
29	8	0	4.575144	-0.917263	1.031365
30	6	0	4.976050	1.265861	-0.425017
31	1	0	4.811526	0.645900	-1.306579
32	1	0	4.551817	2.264473	-0.523489
33	1	0	6.041872	1.316112	-0.191889

TS19.log

Input orientation:

Center Number	Atomic Number	Atomic Type	Coordinates (Angstroms) X	Y	Z
1	6	0	4.712777	-0.219271	0.143181
2	6	0	3.690497	0.220581	0.975146
3	6	0	2.639062	0.979526	0.457053
4	6	0	2.646049	1.328375	-0.894859
5	6	0	3.663180	0.879206	-1.729627
6	6	0	4.691529	0.096002	-1.213282
7	1	0	5.522063	-0.811562	0.553258
8	1	0	3.704220	-0.025781	2.030837
9	1	0	1.861222	1.958320	-1.298344
10	1	0	3.654330	1.146601	-2.779578
11	1	0	5.483557	-0.255579	-1.864049
12	6	0	0.258913	0.596071	1.235636
13	8	0	-0.502966	0.563283	2.271119
14	17	0	1.256536	-1.790715	2.091630
15	6	0	1.521777	1.415283	1.372802
16	1	0	1.805176	1.299873	2.424293
17	1	0	1.288272	2.476111	1.238358
18	15	0	0.347339	-1.076586	0.233044
19	17	0	1.777466	-2.093843	-0.812477

20	17	0	-0.349982	-0.233861	-1.625782
21	8	0	-0.982972	-1.874669	0.582910
22	16	0	-2.308240	-2.638041	-0.201952
23	8	0	-3.035938	-1.619186	-0.901383
24	8	0	-2.905717	-3.333613	0.901575
25	6	0	-1.451445	-3.729660	-1.287400
26	1	0	-0.774683	-4.337136	-0.686286
27	1	0	-2.225280	-4.347490	-1.748939
28	1	0	-0.941173	-3.135603	-2.045391
29	15	0	-1.188011	2.154101	0.006265
30	17	0	-3.090235	1.569751	-0.346764
31	17	0	-0.728675	3.243272	-1.640603
32	17	0	-1.421032	3.580390	1.442204
33	1	0	-1.251249	-0.071219	2.240620

TS2.log

Input orientation:

Center Number	Atomic Number	Atomic Type	Coordinates (Angstroms) X	Y	Z
1	6	0	-4.530317	-0.031277	-0.841207
2	6	0	-3.215374	-0.241774	-1.230357
3	6	0	-2.273136	-0.636377	-0.277022
4	6	0	-2.638784	-0.813887	1.057139
5	6	0	-3.960809	-0.606602	1.435838
6	6	0	-4.902736	-0.212330	0.490729
7	1	0	-5.266043	0.276948	-1.574182
8	1	0	-2.916527	-0.100070	-2.262109
9	1	0	-1.898012	-1.126049	1.783446
10	1	0	-4.253119	-0.752254	2.468680
11	1	0	-5.931031	-0.046022	0.789944
12	6	0	-0.862135	-0.859879	-0.675951
13	8	0	0.061075	-1.212932	0.252148
14	8	0	-0.726811	-1.332381	-1.918035
15	1	0	0.186276	-1.619867	-2.082527
16	15	0	0.301051	0.626507	-0.145574
17	17	0	0.401262	2.641309	-0.998725
18	17	0	0.118310	1.193472	1.798704
19	8	0	1.883118	0.392755	-0.447251
20	16	0	2.926843	-0.775371	0.121134
21	8	0	2.637368	-0.989575	1.519191
22	8	0	2.857951	-1.883277	-0.801310
23	6	0	4.407444	0.151015	-0.103816
24	1	0	5.222668	-0.508183	0.198537
25	1	0	4.481455	0.409737	-1.159007
26	1	0	4.352738	1.032131	0.533440

TS20.log

Input orientation:

Center Number	Atomic Number	Atomic Type	Coordinates (Angstroms) X	Y	Z
1	6	0	0.461127	4.719433	0.217852
2	6	0	0.277491	3.675097	1.116175
3	6	0	-0.443132	2.539387	0.739882
4	6	0	-1.002188	2.480537	-0.537987
5	6	0	-0.810613	3.519300	-1.441443
6	6	0	-0.072765	4.637906	-1.066016
7	1	0	1.024636	5.594504	0.519159
8	1	0	0.695706	3.737282	2.114642
9	1	0	-1.587778	1.620723	-0.839584
10	1	0	-1.240941	3.453437	-2.433647
11	1	0	0.077920	5.448228	-1.769594
12	6	0	0.259001	0.201932	1.430427
13	8	0	0.463716	-0.593053	2.411725
14	17	0	2.644945	1.371623	1.892238
15	6	0	-0.602093	1.404403	1.721017
16	1	0	-0.309439	1.710040	2.732066
17	1	0	-1.645785	1.081802	1.806252
18	15	0	1.724662	0.403399	0.152799
19	17	0	2.443693	1.914421	-1.018800
20	17	0	0.647136	-0.358163	-1.547155
21	8	0	2.704559	-0.834555	0.341832
22	16	0	3.325717	-2.168662	-0.539727
23	8	0	2.206909	-2.975201	-0.934721
24	8	0	4.294494	-2.673181	0.391205
25	6	0	4.088855	-1.342194	-1.894543
26	1	0	4.781485	-0.604760	-1.489616
27	1	0	4.625185	-2.120604	-2.441898
28	1	0	3.311796	-0.905040	-2.520564
29	15	0	-1.473722	-1.332030	0.399650
30	17	0	-0.994114	-3.199186	-0.221268
31	17	0	-2.626835	-1.709180	2.056946
32	1	0	1.067663	-1.350374	2.257698
33	8	0	-2.552026	-0.927116	-0.743259
34	16	0	-3.960871	-0.013084	-0.747400
35	8	0	-3.953764	0.595243	-2.049874
36	8	0	-3.908888	0.809132	0.436957
37	6	0	-5.163761	-1.292089	-0.629326
38	1	0	-6.135122	-0.801620	-0.716610
39	1	0	-4.991349	-1.979556	-1.456266
40	1	0	-5.051149	-1.776485	0.339950

TS21.log

Input orientation:

Center Number	Atomic Number	Atomic Type	Coordinates (Angstroms) X	Y	Z
1	6	0	4.709298	-1.727454	-0.433329
2	6	0	3.492252	-1.189403	-0.843331
3	6	0	3.125612	0.101045	-0.460052

4	6	0	3.993494	0.846245	0.340847
5	6	0	5.207808	0.308451	0.754350
6	6	0	5.568319	-0.980718	0.368409
7	1	0	4.985949	-2.729769	-0.740563
8	1	0	2.823302	-1.772570	-1.467610
9	1	0	3.716305	1.852710	0.636096
10	1	0	5.874599	0.897529	1.374078
11	1	0	6.516214	-1.398871	0.688145
12	6	0	0.793107	0.702365	0.243208
13	8	0	0.943671	1.270327	1.310108
14	17	0	0.215641	-1.248775	0.448180
15	15	0	-3.572086	-0.285788	-0.218485
16	17	0	-3.037290	-0.356103	1.793093
17	17	0	-3.059558	-2.210830	-0.798137
18	8	0	-2.264483	0.506552	-0.844601
19	15	0	-0.987399	1.418359	-0.500456
20	8	0	-0.937721	2.436948	-1.720134
21	1	0	-0.586328	2.081694	-2.554619
22	8	0	-1.467370	2.333840	0.693087
23	1	0	-0.821136	2.293496	1.426476
24	6	0	1.808109	0.688705	-0.895668
25	1	0	1.393112	0.147267	-1.747035
26	1	0	1.949085	1.738564	-1.176568

TS22.log

Input orientation:

Center Number	Atomic Number	Atomic Type	Coordinates (Angstroms) X	Y	Z
1	6	0	3.427605	3.098043	0.106948
2	6	0	2.065359	2.876810	-0.075189
3	6	0	1.389755	1.940452	0.712130
4	6	0	2.101239	1.240646	1.690555
5	6	0	3.460104	1.470344	1.880521
6	6	0	4.128281	2.395849	1.084120
7	1	0	3.939136	3.830922	-0.507172
8	1	0	1.517583	3.441691	-0.819817
9	1	0	1.583531	0.514590	2.309108
10	1	0	3.997298	0.925638	2.649170
11	1	0	5.187769	2.575321	1.229812
12	6	0	-0.487862	1.194714	-0.845962
13	8	0	-0.085762	1.799477	-1.911454
14	15	0	-3.466313	-1.097288	1.160717
15	17	0	-5.283680	-0.874898	0.163420
16	17	0	-3.555736	0.572266	2.406701
17	8	0	-2.449745	-0.538083	0.001833
18	15	0	-2.286104	0.771647	-0.963089
19	8	0	-2.387774	-0.044112	-2.351541
20	1	0	-2.133332	0.466043	-3.139324
21	8	0	-3.242291	1.865096	-0.750474
22	6	0	-0.091813	1.712385	0.540053
23	1	0	-0.633114	2.659518	0.650122

24	1	0	-0.458109	1.029767	1.313448
25	15	0	2.838494	-2.196006	-0.290929
26	17	0	3.209827	-2.422017	1.730023
27	17	0	4.201360	-0.701731	-0.742486
28	8	0	1.478447	-1.242679	-0.077871
29	15	0	0.412572	-0.684595	-1.132859
30	8	0	1.215434	-0.252207	-2.414576
31	1	0	0.906024	0.709403	-2.542056
32	8	0	-0.381503	-2.025284	-1.479819
33	1	0	-1.237087	-1.883929	-1.918888

TS3.log

Input orientation:

Center Number	Atomic Number	Atomic Type	Coordinates (Angstroms) X	Y	Z
1	6	0	-4.258006	0.220536	-0.547568
2	6	0	-3.062235	0.826054	-0.894663
3	6	0	-1.875152	0.326966	-0.330699
4	6	0	-1.869422	-0.747266	0.560866
5	6	0	-3.076138	-1.331895	0.896011
6	6	0	-4.263404	-0.851579	0.341136
7	1	0	-5.184181	0.587901	-0.970917
8	1	0	-3.029579	1.662362	-1.582063
9	1	0	-0.952826	-1.110807	1.007507
10	1	0	-3.093886	-2.163748	1.587921
11	1	0	-5.203339	-1.320425	0.607445
12	6	0	-0.704401	1.025339	-0.765303
13	8	0	-0.265108	1.879742	-1.378109
14	17	0	0.507515	1.553514	1.818794
15	15	0	1.246813	-0.205048	-0.105041
16	17	0	1.515747	-1.603297	-1.682217
17	17	0	2.788805	1.048222	-0.573148
18	17	0	1.984111	-1.376464	1.400801

TS4.log

Input orientation:

Center Number	Atomic Number	Atomic Type	Coordinates (Angstroms) X	Y	Z
1	6	0	-4.238894	0.669145	0.083015
2	6	0	-2.882967	0.583809	0.360821
3	6	0	-2.207346	-0.609834	0.103799
4	6	0	-2.875978	-1.712774	-0.429594
5	6	0	-4.236184	-1.617811	-0.698496
6	6	0	-4.914943	-0.430151	-0.445251
7	1	0	-4.771082	1.589356	0.289438
8	1	0	-2.372655	1.424988	0.814419
9	1	0	-2.331657	-2.626393	-0.635055

10	1	0	-4.761666	-2.470156	-1.111460
11	1	0	-5.975403	-0.358988	-0.656415
12	6	0	-0.752079	-0.770269	0.310602
13	8	0	-0.082225	-1.755207	0.218719
14	17	0	-0.406893	0.141249	2.471317
15	15	0	0.365878	0.766284	-0.245788
16	17	0	0.408499	0.765962	-2.270739
17	17	0	0.147063	2.690598	0.245007
18	8	0	1.847032	0.448277	0.212571
19	16	0	2.928752	-0.852047	-0.008061
20	8	0	2.881170	-1.595473	1.218798
21	8	0	2.575440	-1.471224	-1.257920
22	6	0	4.378987	0.135935	-0.136900
23	1	0	5.207782	-0.563853	-0.256784
24	1	0	4.262409	0.772512	-1.012932
25	1	0	4.471798	0.709000	0.784295

TS5.log

Input orientation:

Center Number	Atomic Number	Atomic Type	Coordinates (Angstroms) X	Y	Z
1	6	0	-3.108547	-2.158216	0.993653
2	6	0	-1.805404	-1.739548	0.761918
3	6	0	-1.541532	-0.880603	-0.305805
4	6	0	-2.565364	-0.443290	-1.141455
5	6	0	-3.865785	-0.881306	-0.911477
6	6	0	-4.138119	-1.731879	0.155518
7	1	0	-3.321539	-2.819587	1.824778
8	1	0	-0.993962	-2.072262	1.400353
9	1	0	-2.346869	0.228577	-1.963132
10	1	0	-4.665554	-0.553536	-1.564588
11	1	0	-5.153684	-2.063938	0.336670
12	6	0	-0.142405	-0.432534	-0.540312
13	8	0	0.054485	0.519801	-1.606187
14	8	0	0.672570	-1.567349	-0.609383
15	16	0	2.282167	-1.571643	-0.300812
16	8	0	2.734546	-0.203322	-0.490180
17	8	0	2.794525	-2.615101	-1.154177
18	6	0	2.369830	-2.045162	1.411004
19	1	0	3.047957	-2.895553	1.468583
20	1	0	2.755563	-1.191468	1.964328
21	1	0	1.368730	-2.318019	1.743525
22	15	0	0.395216	1.242585	-0.140024
23	17	0	-1.262083	2.359408	0.191253
24	17	0	1.161952	1.262455	1.858747
25	17	0	1.739679	2.593323	-0.872086

TS6.log

Input orientation:

Center Number	Atomic Number	Atomic Type	Coordinates (Angstroms) X	Y	Z
1	6	0	4.465634	-0.089026	0.451479
2	6	0	3.141691	0.323848	0.507927
3	6	0	2.260203	-0.063777	-0.502977
4	6	0	2.683515	-0.864545	-1.557008
5	6	0	4.016630	-1.260321	-1.614836
6	6	0	4.904224	-0.874893	-0.613456
7	1	0	5.155805	0.202729	1.234281
8	1	0	2.783917	0.937444	1.328756
9	1	0	1.974332	-1.179456	-2.313870
10	1	0	4.361057	-1.873713	-2.438899
11	1	0	5.939150	-1.193474	-0.658525
12	6	0	0.821333	0.366868	-0.437397
13	8	0	-0.085320	-0.170888	-1.263766
14	8	0	0.856894	1.780356	-0.312229
15	16	0	-0.524599	2.633376	-0.183197
16	8	0	-1.354675	1.910809	0.769218
17	8	0	-1.057950	2.885465	-1.504766
18	6	0	0.137426	4.103317	0.518485
19	1	0	-0.707424	4.782603	0.642443
20	1	0	0.585933	3.845863	1.476616
21	1	0	0.866981	4.511288	-0.180035
22	15	0	-0.174250	-0.670155	0.724621
23	17	0	0.493613	-2.551857	0.493531
24	17	0	-0.021810	-0.414716	2.772765
25	8	0	-1.765961	-0.741511	0.665482
26	16	0	-2.855295	-1.215775	-0.533818
27	8	0	-2.207763	-2.224040	-1.332563
28	8	0	-4.011297	-1.557043	0.253246
29	6	0	-3.110678	0.289084	-1.409724
30	1	0	-3.929091	0.076430	-2.101267
31	1	0	-3.397042	1.046783	-0.681534
32	1	0	-2.191356	0.532840	-1.936083

TS7.log

Input orientation:

Center Number	Atomic Number	Atomic Type	Coordinates (Angstroms) X	Y	Z
1	6	0	1.670980	3.558819	-0.822409
2	6	0	1.089799	2.378289	-1.243764
3	6	0	0.390803	1.581535	-0.315292
4	6	0	0.297017	1.976838	1.033439
5	6	0	0.882847	3.162023	1.436219
6	6	0	1.568662	3.949756	0.512856
7	1	0	2.200932	4.179392	-1.533617
8	1	0	1.154723	2.071323	-2.279695
9	1	0	-0.193352	1.364020	1.779505
10	1	0	0.814913	3.467403	2.472229

11	1	0	2.028144	4.875927	0.837598
12	6	0	-0.173092	0.330729	-0.787948
13	8	0	-0.041159	0.079627	-2.054928
14	17	0	-0.874975	-1.275487	1.670791
15	15	0	-1.858128	-0.227193	0.023932
16	17	0	-2.767540	0.954433	-1.554580
17	17	0	-2.658509	-1.945425	-0.669928
18	17	0	-2.953478	0.804650	1.395791
19	15	0	1.600600	-1.097731	-0.008183
20	17	0	2.493228	-0.526950	1.717059
21	17	0	2.995324	-0.629486	-1.407779
22	17	0	1.654658	-3.122258	0.047516
23	1	0	-0.438297	-0.745980	-2.390811

TS8.log

Input orientation:

Center Number	Atomic Number	Atomic Type	Coordinates (Angstroms) X	Y	Z
1	6	0	3.448017	-2.613417	-1.182604
2	6	0	2.449505	-1.701837	-1.468903
3	6	0	1.935934	-0.896089	-0.434251
4	6	0	2.414310	-1.033153	0.882718
5	6	0	3.411176	-1.952126	1.150487
6	6	0	3.927410	-2.738619	0.121328
7	1	0	3.855988	-3.228130	-1.974794
8	1	0	2.074339	-1.591805	-2.478231
9	1	0	1.997694	-0.463743	1.704790
10	1	0	3.777767	-2.065616	2.162360
11	1	0	4.708274	-3.458024	0.337932
12	6	0	0.861089	0.020594	-0.760218
13	8	0	0.419659	0.000514	-1.971102
14	17	0	-0.327361	1.076522	1.824070
15	15	0	0.900977	1.761263	0.141391
16	17	0	2.190470	2.315517	-1.512277
17	17	0	-0.441252	3.198161	-0.308506
18	17	0	2.348711	2.310293	1.458543
19	1	0	-0.313278	0.605400	-2.204161
20	6	0	-3.963956	1.177937	0.915222
21	1	0	-4.598983	1.915297	0.420017
22	1	0	-4.538872	0.562749	1.606718
23	1	0	-3.108368	1.648361	1.396725
24	16	0	-3.368884	0.126445	-0.360508
25	8	0	-2.510286	-0.973838	0.558101
26	8	0	-4.430302	-0.618538	-0.979803
27	8	0	-2.367881	0.771729	-1.189571
28	15	0	-0.952794	-1.357317	0.226097
29	17	0	-0.543506	-2.446990	1.876019
30	17	0	-1.148271	-2.768089	-1.238220

TS9.log

Input orientation:

Center Number	Atomic Number	Atomic Type	Coordinates (Angstroms) X	Y	Z
1	6	0	-3.513601	2.266649	0.589831
2	6	0	-2.272139	1.657138	0.595725
3	6	0	-1.760501	1.112469	-0.597175
4	6	0	-2.517820	1.174669	-1.784185
5	6	0	-3.758783	1.783019	-1.770785
6	6	0	-4.256878	2.328014	-0.587505
7	1	0	-3.905445	2.687876	1.506575
8	1	0	-1.733617	1.598281	1.532124
9	1	0	-2.121296	0.762136	-2.702748
10	1	0	-4.338507	1.836439	-2.683513
11	1	0	-5.230772	2.803124	-0.583083
12	6	0	-0.469913	0.441615	-0.662340
13	8	0	-0.095477	0.043382	-1.840667
14	17	0	0.204900	-0.019517	2.261242
15	15	0	0.952928	1.046934	0.515470
16	17	0	1.681265	2.200799	-1.170282
17	17	0	0.899126	2.766904	1.606724
18	8	0	2.226549	0.074654	0.575176
19	16	0	3.313469	-0.661796	-0.501842
20	8	0	3.716415	-1.828898	0.224270
21	8	0	2.558053	-0.800455	-1.730549
22	6	0	4.616147	0.516898	-0.596214
23	1	0	5.428855	0.004022	-1.115526
24	1	0	4.268344	1.381731	-1.155746
25	1	0	4.894934	0.762521	0.428688
26	15	0	-1.147195	-1.688888	0.156683
27	17	0	-1.999344	-2.441626	-1.523120
28	17	0	-2.678777	-1.694305	1.476442
29	17	0	0.085476	-3.179150	0.763285
30	1	0	0.801688	-0.359059	-1.917367

benzidronic acid.log

Input orientation:

Center Number	Atomic Number	Atomic Type	Coordinates (Angstroms) X	Y	Z
1	6	0	-3.698092	0.865877	-0.027653
2	6	0	-2.496603	0.951633	0.678757
3	6	0	-1.661202	-0.164287	0.811747
4	6	0	-2.072280	-1.381644	0.256049
5	6	0	-3.273582	-1.471260	-0.444584
6	6	0	-4.085282	-0.346091	-0.596521
7	1	0	-4.330755	1.743038	-0.122391
8	1	0	-2.208055	1.894481	1.139832
9	1	0	-1.453379	-2.266695	0.379964
10	1	0	-3.578658	-2.422967	-0.868962

11	1	0	-5.019897	-0.417904	-1.144043
12	6	0	0.937481	0.096914	0.736235
13	8	0	1.965610	0.284125	1.697325
14	6	0	-0.357267	-0.045802	1.566088
15	1	0	-0.388964	0.838058	2.212264
16	1	0	-0.210852	-0.918710	2.210156
17	15	0	0.988741	1.578189	-0.381209
18	8	0	2.317684	1.667264	-1.046378
19	15	0	1.348544	-1.388985	-0.285421
20	1	0	2.840235	0.250798	1.275455
21	8	0	0.593174	-1.491584	-1.563846
22	8	0	2.941820	-1.322518	-0.458319
23	1	0	3.229283	-0.742960	-1.186931
24	8	0	1.169643	-2.560234	0.773249
25	1	0	1.348314	-3.455017	0.433342
26	8	0	-0.183374	1.493229	-1.456779
27	1	0	-1.091814	1.379237	-1.117635
28	8	0	0.724290	2.839985	0.557337
29	1	0	-0.185699	2.963519	0.880404

----

benzilCOCl_DMSO.log

Input orientation:

Center Number	Atomic Number	Atomic Type	Coordinates (Angstroms) X	Y	Z
1	6	0	-5.515485	0.587400	-0.933434
2	6	0	-4.263078	0.016748	-1.146087
3	6	0	-3.467285	-0.353728	-0.064032
4	6	0	-3.934563	-0.146355	1.234953
5	6	0	-5.184297	0.426565	1.447770
6	6	0	-5.976831	0.795043	0.363153
7	1	0	-6.129141	0.868933	-1.781632
8	1	0	-3.901363	-0.144119	-2.156385
9	1	0	-3.321419	-0.438466	2.082336
10	1	0	-5.539735	0.582976	2.459888
11	1	0	-6.951490	1.239964	0.529398
12	6	0	-1.008617	-0.027369	0.147960
13	8	0	-0.217546	-0.163744	1.010227
14	17	0	-0.974965	1.505766	-0.831649
15	6	0	-2.101293	-0.966944	-0.283786
16	1	0	-1.947196	-1.193046	-1.341129
17	1	0	-1.975602	-1.879279	0.301905

----

benzilCOOH_DMSO.log

Input orientation:

Center Number	Atomic Number	Atomic Type	Coordinates (Angstroms) X	Y	Z
1	6	0	4.455424	-1.850291	0.571383

2	6	0	3.167311	-1.417633	0.278621
3	6	0	2.962779	-0.292483	-0.525531
4	6	0	4.070417	0.391192	-1.030292
5	6	0	5.362274	-0.040693	-0.735603
6	6	0	5.556409	-1.161241	0.064905
7	1	0	4.601915	-2.725929	1.193649
8	1	0	2.309993	-1.953312	0.674788
9	1	0	3.918594	1.263817	-1.658297
10	1	0	6.213623	0.497382	-1.136775
11	1	0	6.560764	-1.499492	0.292845
12	6	0	0.946538	1.006991	0.333050
13	8	0	1.789719	1.651124	1.154152
14	6	0	1.562663	0.197349	-0.796175
15	1	0	0.881151	-0.630296	-0.994954
16	1	0	1.548881	0.853974	-1.672504
17	8	0	-0.238966	1.119624	0.490057
18	1	0	2.709661	1.446410	0.926692

Cl2POPOH2_DMSO.log

Input orientation:

Center Number	Atomic Number	Atomic Type	Coordinates (Angstroms) X	Y	Z
1	15	0	3.550232	-0.134435	0.115841
2	17	0	3.277650	0.880513	1.939505
3	17	0	4.264158	1.460711	-1.028080
4	8	0	2.017966	-0.095396	-0.426242
5	15	0	0.897019	-1.366305	-0.463711
6	8	0	1.790588	-2.476375	-1.254467
7	1	0	1.576141	-2.528205	-2.196602
8	8	0	1.214288	-2.005372	1.005018
9	1	0	0.587920	-1.721941	1.686163

----

fenidronic acid.log

Input orientation:

Center Number	Atomic Number	Atomic Type	Coordinates (Angstroms) X	Y	Z
1	6	0	3.498342	-0.036835	1.033268
2	6	0	2.134507	-0.136262	1.294452
3	6	0	1.212144	0.042218	0.263560
4	6	0	1.668966	0.315416	-1.030885
5	6	0	3.032575	0.415132	-1.282480
6	6	0	3.951642	0.239097	-0.252093
7	1	0	4.206302	-0.176402	1.841643
8	1	0	1.792613	-0.351327	2.297451
9	1	0	0.965585	0.458307	-1.842675
10	1	0	3.373843	0.632100	-2.287610
11	1	0	5.013876	0.317861	-0.450872

12	6	0	-0.276886	-0.076354	0.557006
13	8	0	-0.466143	-0.239223	1.951508
14	15	0	-0.906620	-1.628293	-0.243385
15	15	0	-1.214780	1.463365	0.114238
16	1	0	-1.411903	-0.190937	2.158727
17	8	0	-0.303986	2.703039	0.528337
18	8	0	-1.271548	1.397393	-1.470238
19	8	0	-0.277898	-2.835909	0.346241
20	8	0	-0.747309	-1.508764	-1.824088
21	8	0	-2.505857	1.536814	0.840270
22	8	0	-2.469325	-1.446237	-0.023531
23	1	0	-1.732204	2.123600	-1.920391
24	1	0	0.057881	-1.909843	-2.187221
25	1	0	-3.028332	-2.194207	-0.287116
26	1	0	0.503035	2.850596	0.010931

H2O_DMSO.log

Input orientation:

Center Number	Atomic Number	Atomic Type	Coordinates (Angstroms) X	Y	Z
1	8	0	2.600991	1.751266	-1.996357
2	1	0	3.427239	2.227507	-2.126652
3	1	0	2.872958	0.860685	-1.752702

H2O_H2O.log

Input orientation:

Center Number	Atomic Number	Atomic Type	Coordinates (Angstroms) X	Y	Z
1	8	0	-2.273089	0.619975	1.483643
2	1	0	-2.887575	1.360226	1.522537
3	1	0	-1.402711	1.031136	1.505246

HCl_DMSO.log

Input orientation:

Center Number	Atomic Number	Atomic Type	Coordinates (Angstroms) X	Y	Z
1	17	0	0.000000	0.000000	0.071667
2	1	0	0.000000	0.000000	-1.218333

HCl_H2O.log

Input orientation:

Center Number	Atomic Number	Atomic Type	Coordinates (Angstroms) X	Y	Z
1	17	0	-2.267850	0.643549	1.484882
2	1	0	-3.557850	0.643549	1.484882

MSA_H2O.log

Input orientation:

Center Number	Atomic Number	Atomic Type	Coordinates (Angstroms) X	Y	Z
1	8	0	-0.518241	-0.751862	1.240635
2	8	0	-0.516448	-0.756669	-1.238542
3	8	0	-0.570994	1.401562	-0.003060
4	16	0	-0.089714	-0.125999	0.000133
5	6	0	1.649435	0.141755	0.000698
6	1	0	1.908687	0.694363	0.902260
7	1	0	2.108572	-0.846984	0.002191
8	1	0	1.909578	0.692029	-0.902044
9	1	0	-1.542564	1.481801	-0.000984

PCl2POH2_DMSO.log

Input orientation:

Center Number	Atomic Number	Atomic Type	Coordinates (Angstroms) X	Y	Z
1	15	0	-0.708798	-0.154538	-0.553427
2	17	0	-1.515499	1.726091	-0.061307
3	17	0	-2.184246	-1.362364	0.299201
4	8	0	0.374929	-0.287617	0.651133
5	15	0	2.056925	-0.099346	0.561250
6	8	0	2.369913	-1.144293	-0.649548
7	1	0	2.686814	-2.001720	-0.332287
8	8	0	2.096893	1.207610	-0.416740
9	1	0	2.220229	2.038449	0.063986

PCl2SO2Me_H2O.log

Input orientation:

Center Number	Atomic Number	Atomic Type	Coordinates (Angstroms) X	Y	Z
1	15	0	1.034434	-0.106614	0.658763
2	17	0	2.276450	-1.453582	-0.285459
3	17	0	1.798570	1.670467	-0.098368
4	8	0	-0.222597	-0.303466	-0.440963

5	16	0	-1.777504	0.098147	-0.107578
6	8	0	-1.728244	1.190909	0.843337
7	8	0	-2.384905	0.302341	-1.398505
8	6	0	-2.368584	-1.374452	0.655989
9	1	0	-3.419587	-1.202613	0.891786
10	1	0	-2.250090	-2.190165	-0.055859
11	1	0	-1.784637	-1.536969	1.562047

PCl3_H2O.log

Input orientation:

Center Number	Atomic Number	Atomic Type	Coordinates (Angstroms) X	Y	Z
1	15	0	-0.000246	-0.001285	0.759659
2	17	0	-1.313987	-1.272474	-0.223493
3	17	0	-0.443721	1.773287	-0.223227
4	17	0	1.757995	-0.499608	-0.223555

PhCOCl_DMSO.log

Input orientation:

Center Number	Atomic Number	Atomic Type	Coordinates (Angstroms) X	Y	Z
1	6	0	1.928155	-1.405978	0.000043
2	6	0	0.566378	-1.129917	0.000057
3	6	0	0.133942	0.197509	0.000017
4	6	0	1.064381	1.243881	-0.000045
5	6	0	2.421369	0.958892	-0.000051
6	6	0	2.853310	-0.366200	-0.000012
7	1	0	2.265988	-2.435234	0.000078
8	1	0	-0.145104	-1.945237	0.000101
9	1	0	0.716831	2.269845	-0.000076
10	1	0	3.141220	1.768631	-0.000102
11	1	0	3.914541	-0.587686	-0.000022
12	6	0	-1.292517	0.591729	0.000029
13	8	0	-1.723434	1.694251	0.000091
14	17	0	-2.479772	-0.774343	-0.000055

PhCOCl_H2O.log

Input orientation:

Center Number	Atomic Number	Atomic Type	Coordinates (Angstroms) X	Y	Z
1	6	0	-1.928580	-1.405032	0.000174
2	6	0	-0.566745	-1.131023	0.000382
3	6	0	-0.132849	0.196272	0.000253

4	6	0	-1.061228	1.244726	-0.000036
5	6	0	-2.418327	0.961321	-0.000288
6	6	0	-2.851812	-0.363388	-0.000178
7	1	0	-2.268143	-2.433356	0.000255
8	1	0	0.143740	-1.947069	0.000587
9	1	0	-0.711272	2.269711	-0.000122
10	1	0	-3.137192	1.771484	-0.000512
11	1	0	-3.913077	-0.583250	-0.000419
12	6	0	1.292551	0.583982	0.000145
13	8	0	1.724625	1.691013	0.000282
14	17	0	2.475934	-0.772163	-0.000280

PhCOOH_DMSO.log

Input orientation:

Center Number	Atomic Number	Atomic Type	Coordinates (Angstroms) X	Y	Z
1	6	0	1.833302	-1.233230	0.000021
2	6	0	0.443436	-1.199292	0.000049
3	6	0	-0.217277	0.030539	0.000042
4	6	0	0.512063	1.220884	0.000007
5	6	0	1.900150	1.181504	-0.000025
6	6	0	2.560420	-0.045519	-0.000017
7	1	0	2.349344	-2.186078	0.000028
8	1	0	-0.123816	-2.121575	0.000077
9	1	0	-0.015824	2.167140	0.000003
10	1	0	2.467544	2.104728	-0.000055
11	1	0	3.644172	-0.075659	-0.000044
12	6	0	-1.702635	0.119837	0.000068
13	8	0	-2.326238	1.152146	0.000008
14	8	0	-2.303899	-1.078590	-0.000051
15	1	0	-3.262981	-0.932128	-0.000089

PhCOOH_H2O.log

Input orientation:

Center Number	Atomic Number	Atomic Type	Coordinates (Angstroms) X	Y	Z
1	6	0	1.830964	-1.234255	0.000103
2	6	0	0.441642	-1.198427	0.000121
3	6	0	-0.217435	0.032555	-0.000029
4	6	0	0.512806	1.222477	-0.000220
5	6	0	1.900817	1.180787	-0.000247
6	6	0	2.559259	-0.047120	-0.000086
7	1	0	2.345510	-2.187517	0.000250
8	1	0	-0.126663	-2.119776	0.000282
9	1	0	-0.010820	2.170859	-0.000339
10	1	0	2.469476	2.102798	-0.000394
11	1	0	3.642639	-0.078670	-0.000101

12	6	0	-1.700539	0.116501	0.000063
13	8	0	-2.325220	1.155770	0.000468
14	8	0	-2.302504	-1.078044	-0.000344
15	1	0	-3.263462	-0.944660	-0.000008

Table S2. Energy (Hartree) and entropy (cal molK^{-1}) values obtained for the computations together with imaginary frequencies (1/s).

E, ZPE, U, H, G and S were computed at the M062X/6-311+G (d,p), while E* at the M062X/6-311++G (3df,3pd) level of theory. G_{corr} is the corrected Gibbs free energy value containing the standard state correction and G* = E* + (G – E). Solvents for those species that were used in MSA and in sulfolane as well, are indicated in parentheses.

ID	E	ZPE	U	H	G	S	E*	G_{corr}	Imag. freq.
PhCOOH (H_2O)	-420.78	-420.67	-420.66	-420.66	-420.71	89.65	-420.81	-420.73	
PhCOCl (H_2O)	-805.14	-805.04	-805.03	-805.02	-805.08	93.72	-805.17	-805.10	
PCl_3 (H_2O)	-1721.99	-1721.98	-1721.98	-1721.98	-1722.02	79.703	-1722.02	-1722.05	
Cl_2POSO_2Me (H_2O)	-1925.48	-1925.43	-1925.42	-1925.42	-1925.47	101.684	-1925.59	-1925.58	
TS1	-2142.70	-2142.58	-2142.57	-2142.57	-2142.63	122.496	-2142.78	-2142.70	-281.37
adduct 1	-2142.71	-2142.59	-2142.57	-2142.57	-2142.64	124.597	-2142.79	-2142.71	
TS2	-2346.21	-2346.04	-2346.01	-2346.01	-2346.09	147.732	-2346.35	-2346.23	-331.93
adduct 2	-2346.21	-2346.04	-2346.02	-2346.02	-2346.1	149.239	-2346.36	-2346.24	
TS3	-2527.08	-2526.98	-2526.96	-2526.96	-2527.03	134.04	-2527.15	-2527.09	-142.67
adduct 3	-2527.09	-2526.99	-2526.97	-2526.97	-2527.04	125.944	-2527.17	-2527.11	
TS4	-2730.58	-2730.43	-2730.4	-2730.4	-2730.49	149.3	-2730.72	-2730.62	-112.53
adduct 4	-2730.6	-2730.44	-2730.41	-2730.41	-2730.5	154.915	-2730.74	-2730.64	
TS5	-2730.57	-2730.41	-2730.39	-2730.38	-2730.46	138.643	-2730.71	-2730.6	-184.25
adduct 5	-2730.57	-2730.41	-2730.39	-2730.39	-2730.47	148.826	-2730.72	-2730.61	
TS6	-2934.07	-2933.86	-2933.83	-2933.83	-2933.92	164.045	-2934.28	-2934.13	-312.39
adduct 6	-2934.08	-2933.87	-2933.84	-2933.84	-2933.93	171.325	-2934.29	-2934.14	
adduct 3 + H^+	-2527.47	-2527.35	-2527.33	-2527.33	-2527.4	131.682	-2527.55	-2527.48	
TS7	-4249.45	-4249.32	-4249.3	-4249.3	-4249.38	154.692	-4249.57	-4249.5	-120.87
bis adduct 1	-4249.45	-4249.33	-4249.3	-4249.3	-4249.39	157.269	-4249.58	-4249.51	
TS8	-4452.95	-4452.77	-4452.74	-4452.74	-4452.84	174.158	-4453.14	-4453.02	-119.22
bis adduct 2	-4452.96	-4452.78	-4452.75	-4452.75	-4452.85	184.027	-4453.15	-4453.04	
adduct 4 + H^+	-2730.98	-2730.8	-2730.78	-2730.78	-2730.86	150.152	-2731.12	-2731.01	
TS9	-4452.96	-4452.78	-4452.75	-4452.75	-4452.85	176.465	-4453.14	-4453.03	-100.02
bis adduct 3	-4452.96	-4452.78	-4452.75	-4452.75	-4452.85	181.742	-4453.15	-4453.04	
TS10	-4656.46	-4656.23	-4656.2	-4656.2	-4656.31	201.258	-4656.71	-4656.55	-109.45
bis adduct 4	-4656.47	-4656.24	-4656.2	-4656.2	-4656.31	204.291	-4656.72	-4656.56	
fenidronic acid (H_2O)	-1482.16	-1481.98	-1481.96	-1481.96	-1482.04	140.816	-1482.29	-1482.15	

fenidronic acid (DMSO)	-1482.15	-1481.96	-1481.94	-1481.94	-1482.02	140.359	-1482.27	-1482.14	
H_2O (H_2O)	-76.4347	-76.4134	-76.4101	-76.409	-76.4347	46.341	-76.4389	-76.4358	
HCl (H_2O)	-460.799	-460.793	-460.79	-460.789	-460.814	45.69	-460.804	-460.816	
$Cl_2POP(OH)_2$	-1830.08	-1830.04	-1830.03	-1830.03	-1830.09	105.587	-1830.15	-1830.16	
PhCOCl (DMSO)	-805.14	-805.041	-805.031	-805.03	-805.083	94.612	-805.172	-805.109	
PhCOOH (DMSO)	-420.78	-420.67	-420.66	-420.659	-420.709	89.311	-420.816	-420.736	
HCl (DMSO)	-460.80	-460.795	-460.795	-460.791	-460.816	45.69	-460.806	-460.818	
TS11	-2250.83	-2250.68	-2250.66	-2250.66	-2250.73	140.926	-2250.94	-2250.83	-163.32
adduct 7	-2250.84	-2250.68	-2250.66	-2250.66	-2250.74	144.773	-2250.95	-2250.84	
TS12	-2635.19	-2635.05	-2635.03	-2635.03	-2635.11	147.979	-2635.3	-2635.21	-119.33
adduct 8	-2635.21	-2635.07	-2635.04	-2635.04	-2635.13	154.024	-2635.31	-2635.23	
TS13	-2635.2	-2635.06	-2635.03	-2635.03	-2635.11	146.382	-2635.3	-2635.21	-163.32
adduct 9	-2635.23	-2635.09	-2635.07	-2635.07	-2635.16	158.798	-2635.34	-2635.26	
adduct 9 - HCl	-2174.42	-2174.29	-2174.27	-2174.27	-2174.35	143.978	-2174.52	-2174.45	
TS14	-4004.49	-4004.32	-4004.29	-4004.29	-4004.4	196.13	-4004.67	-4004.57	-251.78
bis adduct 5	-4004.53	-4004.36	-4004.33	-4004.33	-4004.43	190.521	-4004.72	-4004.61	
BnCOCl (H_2O)	-844.44	-844.313	-844.301	-844.3	-844.357	102.341	-844.47	-844.38	
BnCOCl (DMSO)	-844.45	-844.32	-844.31	-844.31	-844.36	96.10	-844.48	-844.38	
TS15	-2566.40	-2566.26	-2566.24	-2566.24	-2566.32	138.28	-2566.47	-2566.38	-142.82
adduct 10	-2566.41	-2566.27	-2566.25	-2566.25	-2566.33	136.33	-2566.48	-2566.40	
TS16	-2769.89	-2769.70	-2769.68	-2769.68	-2769.76	159.12	-2770.03	-2769.90	-64.34
adduct 11	-2769.90	-2769.72	-2769.69	-2769.69	-2769.78	159.78	-2770.05	-2769.92	
adduct 10 + H^+	-2566.78	-2566.63	-2566.61	-2566.61	-2566.68	132.21	-2566.86	-2566.76	
TS17	-4288.76	-4288.60	-4288.57	-4288.57	-4288.66	162.57	-4288.88	-4288.78	-64.33
bis adduct 6	-4288.76	-4288.61	-4288.58	-4288.58	-4288.67	164.65	-4288.89	-4288.79	
TS18	-4492.25	-4492.04	-4492.01	-4492.01	-4492.11	186.53	-4492.44	-4492.30	-124.43
bis adduct 7	-4492.27	-4492.06	-4492.02	-4492.02	-4492.13	189.19	-4492.46	-4492.32	
adduct 11 + H^+	-2770.28	-2770.08	-2770.06	-2770.06	-2770.14	155.69	-2770.43	-2770.29	
TS19	-4492.26	-4492.05	-4492.02	-4492.02	-4492.12	186.42	-4492.44	-4492.30	-135.46
bis adduct 8	-4492.26	-4492.06	-4492.02	-4492.02	-4492.12	186.22	-4492.45	-4492.31	
TS20	-4695.76	-4695.50	-4695.46	-4695.46	-4695.58	211.65	-4696.01	-4695.83	-145.60
bis adduct 9	-4695.77	-4695.51	-4695.47	-4695.47	-4695.59	210.40	-4696.03	-4695.84	
TS21	-2674.51	-2674.34	-2674.32	-2674.32	-2674.39	146.64	-2674.61	-2674.49	-135.33
adduct 12	-2674.53	-2674.36	-2674.34	-2674.34	-2674.42	153.19	-2674.64	-2674.52	
TS22	-4043.80	-4043.60	-4043.58	-4043.58	-4043.65	167.04	-4043.96	-4043.82	-274.31
bis adduct 10	-4043.84	-4043.64	-4043.61	-4043.61	-4043.70	179.68	-4044.03	-4043.88	
benzidronic acid	-1521.20	-1520.99	-1520.96	-1520.96	-1521.04	145.93	-1521.60	-1521.43	

MSA (H_2O)	-664.30	-664.24	-664.24	-664.23	-664.28	75.77	-664.38	-664.35	
MSA^- (H_2O)	-663.88	-663.83	-663.82	-663.82	-663.86	75.14	-663.95	-663.93	
